# Physically stimulus-responsive nanoparticles for therapy and diagnosis

**DOI:** 10.3389/fchem.2022.952675

**Published:** 2022-09-14

**Authors:** Fatemeh Farjadian, Soheila Ghasemi, Mohsen Akbarian, Mojtaba Hoseini-Ghahfarokhi, Mohsen Moghoofei, Mohammad Doroudian

**Affiliations:** ^1^ Pharmaceutical Sciences Research Center, School of Pharmacy, Shiraz University of Medical Sciences, Shiraz, Iran; ^2^ Department of Chemistry, College of Sciences, Shiraz University, Shiraz, Iran; ^3^ Department of Chemistry, National Cheng Kung University, Tainan, Taiwan; ^4^ Centre de Recherche du Centre Hospitalier de l’Université de Montréal (CRCHUM), Montréal, QC, Canada; ^5^ Department of Microbiology, Faculty of Medicine, Kermanshah University of Medical Sciences, Kermanshah, Iran; ^6^ Department of Cell and Molecular Sciences, Faculty of Biological Sciences, Kharazmi University, Tehran, Iran

**Keywords:** drug delivery, diagnosis, theranostic, micelles, hydrogel, mesoporous silica, magnetic nanoparticles, liposomes

## Abstract

Nanoparticles offer numerous advantages in various fields of science, particularly in medicine. Over recent years, the use of nanoparticles in disease diagnosis and treatments has increased dramatically by the development of stimuli-responsive nano-systems, which can respond to internal or external stimuli. In the last 10 years, many preclinical studies were performed on physically triggered nano-systems to develop and optimize stable, precise, and selective therapeutic or diagnostic agents. In this regard, the systems must meet the requirements of efficacy, toxicity, pharmacokinetics, and safety before clinical investigation. Several undesired aspects need to be addressed to successfully translate these physical stimuli-responsive nano-systems, as biomaterials, into clinical practice. These have to be commonly taken into account when developing physically triggered systems; thus, also applicable for nano-systems based on nanomaterials. This review focuses on physically triggered nano-systems (PTNSs), with diagnostic or therapeutic and theranostic applications. Several types of physically triggered nano-systems based on polymeric micelles and hydrogels, mesoporous silica, and magnets are reviewed and discussed in various aspects.

## Introduction

In the last few years, the increased incidence of chronic diseases such as cancer and metabolic disorders has triggered the need for more efficient, specific, and localized treatments that can deliver drugs at the target site in a highly controlled manner and also allow a precise and early diagnosis (Sahle et al., 2018). Nanotechnology-based systems, or nano-systems, are widely used to address these needs, and various techniques have been developed to obtain more specific and personalized treatments (Farjadian et al., 2019a). Nano-systems in the human body can perform the function of carrying an active substance (drug, contrast agent, and biologic molecules) to a defined site or can constitute themselves the active substance (their imaging or therapeutic properties) (Deng et al., 2020). The design and properties of nano-systems permit combining tissue targeting, molecular diagnosis, cellular imaging, and drug delivery approach to obtain a synergic effect and efficacious responses (Mitra et al., 2017).

Nano-theranostics is a rapidly increasing interest with simultaneous diagnosis and therapy, which resulted in the development of “personalized medicine” (Xie et al., 2010; Zhu et al., 2016; Calatayud et al., 2022). The most important properties to consider for this approach where control drug loading capacity, release, and system stability are size, charge, surface properties, shape, *in vivo* distribution, and toxicity (Wong et al., 2020).

Similarly, nano-systems for diagnostics provide rapid and early disease detection. Several types of nanoparticles, including polymeric micelles and hydrogels, silica, gold, and magnetic nanoparticles, allow us to imagine pathologies and understand the physiological mechanisms of diseases and treatments (Li A. et al., 2021). Nonetheless, their take up in clinical settings has been slow due to the complex pharmacokinetic and pharmacology associated effects (Xie et al., 2010). Several nano-systems can be used as non-invasive contrast agents when paired with a suitable imaging technique. For instance, in whole-body scans, encapsulated nano-contrast agents could be adopted in computed tomography (CT), magnetic resonance imaging (MRI), single-photon emission CT (SPECT), and positron emission tomography (PET) techniques. Conversely, for organ-specific examination, ultrasound, optical imaging (OI), and photoacoustic imaging (PAI) are to be preferred when associated with simpler micro-bubble nano-systems (Sijumon Kunjachan et al., 2012). For imaging purposes, the nano-systems formulation in the size range between 5 and 100 nm permits the acquisition of certain imaging information and allows a rapid and high specific contrast enhancement (Kiessling et al., 2014).

More recently, advanced nano-delivery systems have developed to release the cargo from the carrier at the target site in a temporally and spatially controlled manner while minimizing the side effects of the treatment (Moradi Kashkooli et al., 2020). The optimal nanoparticle sizes for drug delivery systems range between 10 and 100 nm, which are to be exploited for enhanced permeation and retention (EPR) effects (in tumors condition) and to avoid elimination in the spleen (Petros and DeSimone, 2010).

**FIGURE 1:**
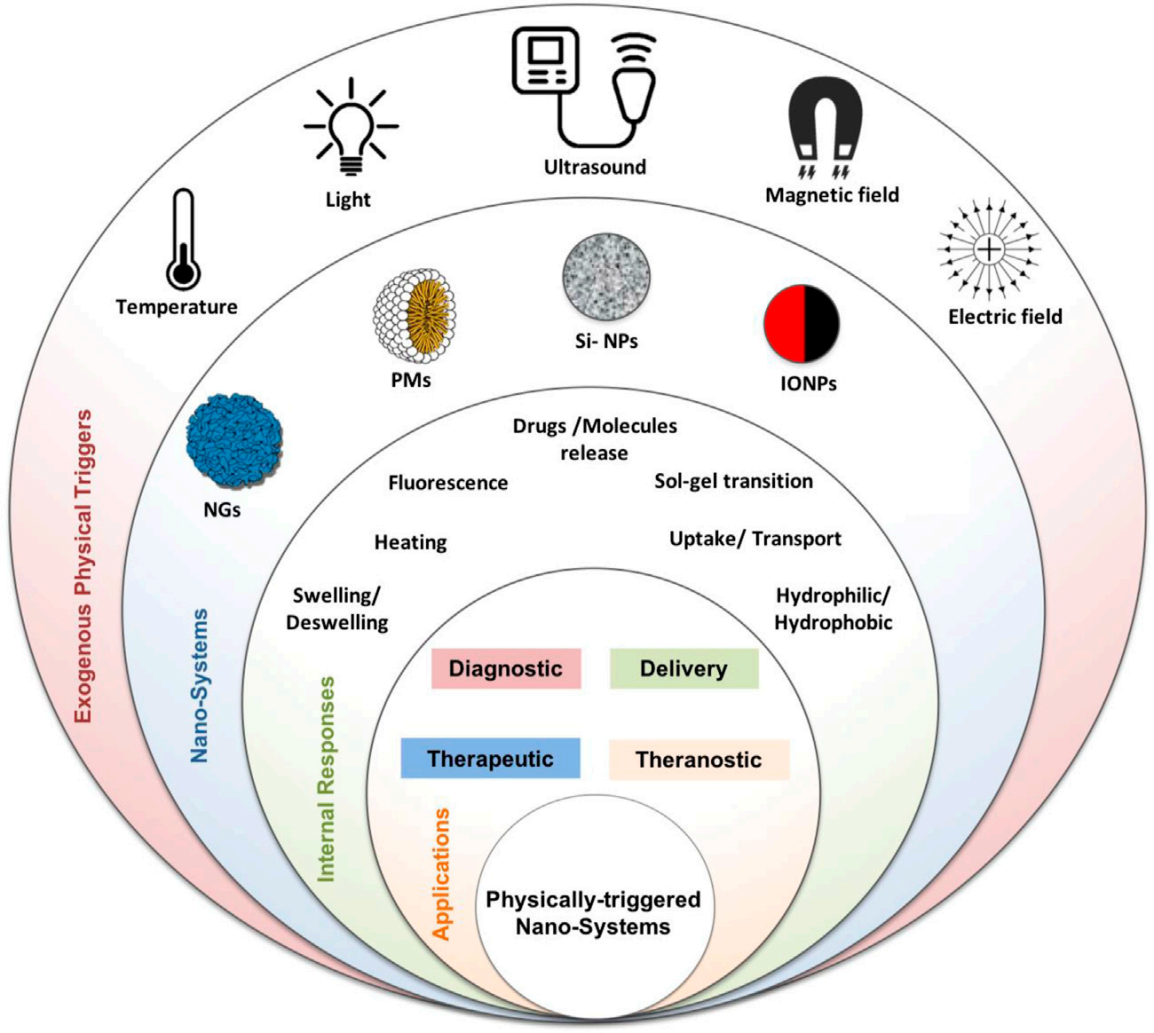
Physically triggered nano-systems (PTNSs) schematic image: exogenous physical triggers (temperature, light, ultrasound, magnetic, and electric fields), nano-systems (nanohydrogel (NGs), polymeric micelle (PMs), silica NPs (Si-NPs), iron oxide magnetic NPs (IONPs)), and their internal response and biomedical applications.

In all cases described earlier, either contrast agents or drugs, various triggers, namely, endogenous or exogenous stimuli, can control the kinetic, release, or enhanced imaging. Endogenous factors include changes in pH, electronic balance, and enzyme concentration. They are used for controlling the drug release and biodistribution from nanocarriers and incrementing more treatment activity at the targeted site (Ahmadi et al., 2020). Exogenous factors are physically induced and include temperature, light, magnetic field, and ultrasound. Unlike endogenous stimuli, which are connected more with disease progression stages, external triggers are controlled and less associated with subject variability (Hosseini et al., 2016; Ghasemiyeh and Mohammadi-Samani, 2021). The choice of using a specific stimulus type is made by considering several factors such as; 1) the pre-designed application, 2) the target site, 3) the expanses, and 4) safety concerns. In combination with imaging agents, exogenous or endogenous stimuli could provide improved platforms for advanced imaging, treatment, or theranostics (Sahle et al., 2018; Li et al., 2019). The physical triggers, nano-systems, and biomedical applications discussed within this review are presented in Figure 1.

### Types of exogeneous physical triggers

Temperature is one of the most applied physical triggers. Polymeric systems with a lower critical solution temperature (LCST) mostly undergo a phase transition when they experience temperature above their LCST. In contrast, other polymeric systems that become soluble over heating have an upper critical solution temperature (UCST) (Schmaljohann, 2006a). Thermo-responsive drug delivery systems (DDSs) offer multiple benefits, sometimes by eliminating the urgent demand for invasive surgery and the delivery to hard-access organs (Fitzpatrick et al., 2012; Lencioni and Cioni, 2016).

Light trigger in the form of UV, visible, or near-infrared (NIR), is usually applied as an excitation source. In the case of converting light to heat, known as photothermal therapy (PTT) or photodynamic therapy (PDT), the light source (i.e., NIR) is applied to kill cancer cells (Hashida et al., 2014; Timor et al., 2015; Hu et al., 2012). PDT induces surrounding oxygen molecules to generate cytotoxic singlet oxygen (^1^O_2_) or reactive oxygen species (ROS), which will be able to destroy cells (Chen Q. et al., 2015; Chen S. et al., 2015).

Alternating magnetic field (AMF) is another source for designing magneto-responsive DDS or gene delivery systems. Magneto-responsive nano-systems can generate heat upon sensing AMF and can be utilized for magnetic hyperthermia treatment. On the other hand, such particles can be used as contrasting agents to provide the signal-to-noise ratio in magnetic resonance imaging (MRI). Magnetic nanoparticles (MNPs) are a suitable carrier for designing therapeutic systems and have been applied as a diagnostic tool for MRI. MNPs have been applied widely in designing theranostic agents, participating in therapy and diagnosis through MRI (Court et al., 2017).

Sonography is a well-known imaging method based on ultrasound waves created by mechanical oscillations of a piezoelectric material when an alternating current exerts. In the last decades, 3D ultrasound imaging has developed to have three-dimensional images and a better concept of organ volume/area, resulting in an advanced diagnosis of abnormalities in the early stages (Huang and Zeng, 2017).

X-ray computed tomography (CT) is a non-invasive clinical imaging modality that combines X-ray images from different body angles with computer processing techniques and provides valuable anatomical information with high spatial resolution.

In continuous of our previous review articles in the field of pharmaceutics (Farjadian et al., 2018; Entezar-Almahdi et al., 2020; Hoseini-Ghahfarokhi et al., 2020; Zarkesh et al., 2021; Hejabi et al., 2022), in this review, we focus on physically triggered nano-systems (PTNS) with diagnostic, therapeutic and theranostic applications. The PTNS cited in this work spans from polymeric micelles (PMs), nanogels, and silica, to MNPs that are responsive to physical stimulus. Each section presents a specific nano-system, and its applications, as evidenced in the literature; the different mechanisms of action and the specific nano-system response are highlighted when subjected to an exogenous physical stimulus.

In the last 10 years, many preclinical studies were performed on PTNS to develop and optimize stable, precise, and selective therapeutic or diagnostic agents. The systems must meet the requirements of efficacy, toxicity, pharmacokinetics, and safety before clinical investigation.

Interestingly, several PTNS have entered clinical studies, and in this review, we are reporting some examples of PTNS currently under investigation.

In the following sections, we will expand on four types of PTNS. Starting from polymeric micelles and nanogels and we will move into mesoporous silica nanoparticles (MSNs) and MNPs. Conclusions and future outlook present an overall view that PTNS has the potential to be used in medical applications.

## Polymeric micelles

Polymeric micelles (PMs) are composed of hydrophilic–hydrophobic segments (amphiphilic diblock, triblock, graft, or either star *co*-polymers) that are self-assembled in aqueous media. At the same time, their concentration is more than critical micelle concentration (CMC). Some procedures have been applied for micelle production like oil-in-water emulsion, solubilization of the copolymer, and subsequent solvent evaporation, dialysis, and film casting. Variously reported morphologies for PMs are spherical, star, worm, crew-cut, flower-like, unimolecular, toroids, helices, cylindrical, lamellae, and vesicles configurations (Topete et al., 2015).

Micelles encapsulating therapeutic macromolecules have been explored for various diseases due to their ability to enhance drug absorption, control the release of the drug at target sites, and prolong the residence time (Xu et al., 2013). This section discusses temperature, light, and ultrasound-responsive PMs with therapeutic and diagnostic applications.

### Polymeric micelles in therapy and diagnosis

The geometrical shapes of PMs depend on external parameters, for example, temperature, solvent, and pH of the medium. These factors significantly affect the length of PMs building blocks. The suitable size of core-corona aggregates of micelles for pharmaceutical applications changed approximately from 10–100 nm (Zhang Y. et al., 2014). Cargos with poor solubility in water can load in the micellar core, and subsequent release can occur through a disintegration procedure. The longer hydrophilic shell (e.g., PEG) extends the micelle stability. It protects the drugs against the external medium, whereas the shorter hydrophobic interior part improves the loading of the lipophilic therapeutic agents such as some drugs, genes, and proteins (Movassaghian et al., 2015; Cabral et al., 2018; Hanafy et al., 2018). Different types of cargo are loaded successfully on the PMs, and their release patterns are investigated (Li N. et al., 2016; Qu et al., 2017). Anticonvulsant drug (clonazepam) (Choi et al., 2006), ophthalmic drugs (e.g., prednisone acetate) (Chang et al., 2008), diazepam (Suksiriworapong et al., 2014), and mainly anticancer drugs, for example, DOX (Panja et al., 2016a), MTX (Tu et al., 2018), PTX (Song et al., 2016), CPT (Meng et al., 2013), curcumin (Yu et al., 2014), cis-Pt (Wang Y. et al., 2017), and ADR (Li et al., 2017). There are different strategies for reaching the drugs to target cells as depicted in Figure 1 (Singh A. et al., 2016). Passive targeting using the EPR effect is the key mechanism and originated from the tendency of nanoparticles (NPs), for example, PMs, for accumulation in tumor cells compared to normal cells. Remaining PMs in tumor tissues for a long time facilitate the sustained release of therapeutic agents into the tumor environment. However, active targeting is based on ligand-mediated targeting and utilizes receptors, for example, FA, HA, carbohydrates, monoclonal antibodies or proteins, and peptides, for example, transferrin, luteinizing hormone, α2-glycoprotein, and aptamer (Amjad et al., 2017; Kesharwani et al., 2018).

**FIGURE 2:**
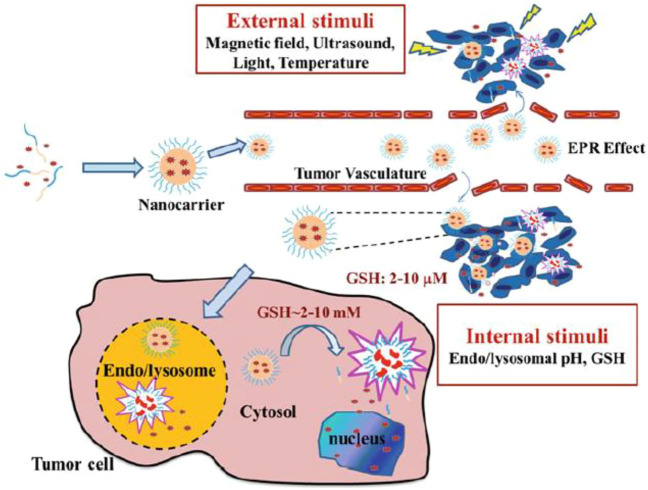
Illustrative description of controlled drug delivery of stimuli-responsive PMs. It is reprinted with permission from Springer (Singh A. et al., 2016).

Charged drug micelles must be designed to inhibit premature drug delivery before encountering the target cells to develop the therapeutic function and lead to site-specific drug delivery while reducing cytotoxicity. In this regard, the targeting process that applied stimuli-responsive nanocarriers for distinct liberation of the drug in the objective cell environment must be engineered (Joglekar and Trewyn, 2013; Nakayama et al., 2014; Zhou Q. et al., 2018; Li Y. et al., 2018). This involves the manipulation of the PMs to respond to definite physical, chemical, or enzymatic triggers that are distinctive to target cells. Figure 2 represents an operating mechanism of internal (pH, temperature, enzyme, ionic strength, and redox potential) or external stimuli (magnetic field, light, and ultrasound) for intelligent drug delivery of PMs (Biswas et al., 2016). Herein, physically triggered PMs (e.g., temperature, light, and ultrasound-triggered) for therapy and diagnosis are discussed. Examples are mentioned in Table 1, regarding the types of stimuli based on structure, cargo, therapy, diagnosis tools, and *in vitro* and *in vivo* assays.

**TABLE 1:** Physically triggered polymeric micelles concerning the type of stimulus and cargo for *in vitro* and *in vivo* investigation, therapy, and diagnosis.

Carrier type based on PMs	Cargo	Physical stimulus/therapy	*In vitro* assay	*In vivo* assay	Imaging mode	Reference
Hydrazine-modified PNIPAAm-*co*-PAA	DOX	Temperature	MCF-7	—	—	Farjadian et al. (2020)
PNIPAAm-*b*-P4VP/silica	DOX	Temperature	—	—	—	Wu et al. (2018a)
(PNIPAAm-*co*-PLys)-X genipin	MTX	Temperature		—	—	Tu et al. (2018)
PEGS-EVOHS-RA	Epirubicin	Temperature	HepG2	—	—	Hassanzadeh et al. (2017)
Poly(diEGMA-co-OEGMA300)-b-PEHMA	Sq GEM and PTX	Temperature	—	—	—	Emamzadeh et al. (2018)
Star (NVCL/NVP-VAc)	MTX	Temperature	—	—	—	Cortez-Lemus and Licea-Claverie, (2017)
PECT	Cognate	Temperature	—	Bcap-37 TBM	—	Huang et al. (2017)
PLA-PNIPAAm-PLA	ADR	Temperature	—	—	—	Li et al. (2017)
PE-PCL-b-PNIPAAm and PE-PCL-b-PNVCL	DOX	Temperature	C6 glioma	C6 glioma TBM	FI	Panja et al. (2016a)
β-CD-PNIPAAm star polymer	PTX	Temperature	AT3B-1	—	—	Song et al. (2016)
PLLA–L35–PLLA	DOC and OXA	Temperature	CT26, HEK293, and 3T3	CRPC-TBM	—	Omlor et al. (2015)
P(MEO_2_ MA-co-OEGMA)-b-PLLA-b-P(MEO2 MA-co-OEGMA	Curcumin	Temperature	—	—	—	Meir et al. (2015)
PECT	DOX and ^131^I-HA	Temperature/radiotherapy	HepG2	HepG2 TBM	FI	Huang et al. (2015)
P(NIPAAM-co-AAm)-b-PBMA	MTX	Temperature	LLC	—	—	Slaughter and Sunday, (2014)
P(NIPAAM-co-DMAAm)-b-PLLA-b-P(NIPAAm-co-DMAAm)	Curcumin	Temperature	L929, A549	—	—	Yu et al. (2014)
Alg-g-PNIPAAm	DOX	Temperature	SCC7	SCC7 TBM	NR FI	Ahn et al. (2014)
mPEG-b-p(HPMAm-Bz/Nt-co-HPMAm-Lac)	PTX and DTX	Temperature	B16F10	—	—	Shi et al. (2013)
PLG-g-PMEOiMA	DOX	Temperature	HeLa	—	—	Ding et al. (2013)
Biotin-PEG-b-P(NIPAAm-co-HMAAm)	MTX	Temperature	HeLa, A549 and ECV304	—	—	Cheng et al. (2008)
Poly(ether urethanes)	DOX	Temperature	HepG2	—	—	Sardon et al. (2015)
BU-PPG	DOX	Temperature	MCF-7		—	Gebeyehu et al. (2019)
(c-PNIPAAm)-b-PCL	DOX	Temperature	HeLa	—	—	Wan et al. (2011)
PMPAAm-b-P(NIPAAm-co-MPAAm)-b-PLA	CM-DiI	Temperature	—	—	—	Akimoto et al. (2018)
TBO-CHI-PPS (TCP)	Thymol	Light/PDT	—	—	—	Wang et al. (2019b)
(PPS-P(NIPAM-co-DMAAm))	DOX and ICG	Light/PDT	A549	A549 TBM	FI	Zheng et al. (2018a)
PEG-PLys	BDPI	Light/PDT	HepG2	EMT6 TBM	NIR FI	Ruan et al. (2018)
Oleyl hyaluronan	Cypate	Light/PTT	NIH-3T3	4T1 TBM	NIR FI	Achbergerová et al. (2018)
PEG-b-P(NAGA-co-AN)	DOX and IR780	Light/PTT	MCF-7	MCF-117/DOX TBM	NIR FI	Deng et al. (2018)
PEG-b-PCL-b- PPEMA	Cypate and DPAE	Light/PTT and PDT	4T1	4T1 TBM	—	Zhou et al. (2018a)
mPEG-Azo-PAsp-IM	Ce6	Light/PDT	LLC	LLC TBM	FI	Zhang et al. (2018b)
PCL-b-P(TEGMA-co-NMFA) & PCL-b-PDEGMA	ICG	Light/PDT and PTT	HeLa	—	—	Chien et al. (2018)
PAMD-Ch	siRNA and IR780	Light/PTT	—	—	—	Chen et al. (2018)
Cholesterol-PEG	PpIX	Light/PDT	A549	U14 TBM	Confocal FI	Lin et al. (2018)
mPEG-b-Plys	DOX and ICG	Light/PTT	HeLa	—	—	Li et al. (2018d)
pHPMA	PyF	Light/PDT	C26 and melanoma B16-F10	S180 cells, C26 cells, and B16-F10 cells	FI	Fang et al. (2018)
HA-b-PLGA	PpIX	Light/PDT	A549	—	—	Li et al. (2018c)
PFOC-PEI-M	Ce6	Light/PDT	C6 glioma	C6 glioma TBM	FI	Wang et al. (2018c)
PEG-PSDEA-PEG	SN38	Light/PDT	BNL 1MEA.7R.1murine carcinoma	BNL 1MEA.7R.1 TBM	—	Yang et al. (2018a)
PS70.5-b-PAA13	ClAlPc	Light/PDT	Caco-2	—	—	Vilsinski et al. (2018)
PEG-based micelle	AIE-1	Light/PDT	HeLa	—	—	Zhang et al. (2018c)
PEG-b-PCPH	[Ru(CHLtpy)(biq)(H_2_O)]^2+^	Light/PDT	HeLa	HeLa TBM	TI	Sun et al. (2018c)
AC-CS	DOX and PpIX	Light/PDT	MCF-7/ADR	MCF-7/ADR TBM	—	Wei et al. (2018b)
Se-based PMs	DOX and ICG	Light/PDT	4T1	4T1 TBM	—	Huang et al. (2017)
DEACM-PEG	—	Light/PTT	Renca	—	—	Zhang et al. (2017)
(PEO-b-PBLG)-NO	Platinum (IV) prodrug	Light/PTT	HCT-116 and MCF-7	—	—	Pramanick et al. (2017)
POEGMA-b-P(NIPAM-co-NBA-co- Gd	DOX	Light/PTT	HepG2	—	MRI	Li et al. (2012)
HPHEEP-DNQ	C 102	Light/PTT	HepG2 and HUVEC	—	—	Chen et al. (2011)
POEGMA-b-PFMA	DOX and ICG	Light/PTT	HeLa	—	—	Li et al. (2015)
PEG-b-PC-SP	C 102	Light/PTT	HeLa	—	—	Hu et al. (2015b)
EC-g-PHEMA-g-PSPMA	Pyrene	Light/PTT	—	—	—	Wang et al. (2014)
PEG:C10-PMA	IBSP	Light/PTT	HeLa	—	—	Aibani et al. (2017)
PLL-g-PEG/DNQ	DOX and PFTTQ	Light/PTT	MDA-MB-231, MCF-7 and 293T	—	FI	Yuan et al. (2015)
PEG-NP	NR	Light	—	—	—	Zhang et al. (2014b)
mPEG-b-poly(Tyr)-g-NBA	NR	Light	—	—	—	Hu et al. (2015b)
PEG-b-PC-Azo	NR	Light	HeLa	—	—	Hu et al. (2014)
mPEG-b-poly(Tyr)-SP	C 102	Light	HeLa	—		Niu et al. (2014b)
Poly(2-oxazoline)-based micelles	Dexamethasone	Ultrasound	—	—	—	Salgarella et al. (2018)
siRNA micelle-NBs	PTX and siRNA	Ultrasound	HepG2	HepG2 TBM	-	Yin et al. (2014)
siRNA micelle-NBs	siRNA	Ultrasound	C6	Xenograft C6 glioma TBM	USI	Yin et al. (2013)
PLA-b-PEG	NR	Ultrasound	—	—	—	Zhang et al. (2009)
Pluronic, plurogel, and PNHL	DOX	Ultrasound	—	—	—	Husseini et al. (2007)
PEG-b-PPG	NR	Ultrasound	—	—	—	Li et al. (2016a)
Pluronic P-105	DOX	Ultrasound	HL-60, MDR, A2780, A2780/ADR, and MCF-7	—	—	Marin et al. (2002); Rapoport (2004)
P(MEO2MA-*co*-MEO3MA-co-SPMA) & P(MEO2MA-*co*-MEO3MA)-*b*-PSPMA	DOX	Temperature and light	—	—	—	Yang et al. (2019)
PNIPAAm-b-PNBM	NR	Light and temperature	—	—	—	Yang et al. (2015)
PEG(-b-PNBM)-b-PNIPAAm	NR	Light and temperature	—	—	—	Huo et al. (2017)
DDND	miR-345 and GEM	Temperature and pH	Capan-1 and CD18/HPAF	Xenograft TBM	—	Uz et al. (2019)
Sulfonamide-functionalized PNIPAAm	PTX	Temperature and pH	—	—	—	Cyphert et al. (2018)
HAPs-g-PCL-b-PNIPAAm	DTX	Temperature and pH	MCF-7	—	—	Ghamkhari et al. (2018)
β-CD-PNIPAAm and BM-PCL	DOX	Temperature and pH	Hela	—	—	Zhou et al. (2018b)
PNIPAAm-a-PXCLs	DOX	Temperature and pH	HeLa	—	—	Lin et al. (2017)
CPiPrOx-b-PAA	DOX	Temperature and pH	4T1, BGC823, and NIH 3T3	H22 TBM	—	Chen et al. (2017c)
PMMA-b-P[MAA-co-DEGMA]	DOX	Temperature and pH	A2780	—	—	Khine et al. (2015)
PCL-b-P(TEGMA-co-NMFA	DOX	Temperature and pH	HeLa and HT-29	—	—	Wu et al. (2014)
CS-g-PNIPAAm and ALG-g-P(NIPAM-co-NVP)	5-FU	Temperature and pH	Hela	—	—	Ding et al. (2013)
PID_118_-b-PLA_59_ and PID_118_ -b-PCL_60_	ADR	Temperature and pH	N-87	—	—	Jain et al. (2011)
CSO-g-Pluronic	DOX	Temperature and pH	—	—	—	Yang et al. (2010)
PNIPAAm-b-PHpr	Indomethacin	Temperature and pH	—	—	—	Lee et al. (2010)
PHCS-g-PNIPAAm and P(AA-co-tBA)	Prednisone acetate	Temperature and pH	—	—	—	Jia et al. (2010)
PCL-g-(HEMA-co-NIPAM-co-AA)	DOX	Temperature and pH	—	—	—	He et al. (2010)
P(NIPAAm-*co*-AA)-*b*-PCL	PTX	Temperature and pH	HepG2	—	—	Zhang et al. (2007)
PNVIm-PNIPAAm	BSA	Temperature and pH	MCF-7	—	—	Kang et al. (2018)
PNIPAAm-*b*-MAA	Cis-Pt	Temperature and pH	7F2 osteoblast-like	—	—	Wang et al. (2017b)
POSS/PDMAEMA-*b*-PNIPAAm	PTX	Temperature and pH	HUVECs and B16F10		-	Yang et al. (2016c)
PGMA-g-(PS-r-PDMAEMA-r-POEGMA)	DOX and Rh	Temperature and pH	—	—	—	Li et al. (2016d)
LbL films PMs of PDMA-b-PDEA and PSS	Pyrene	Temperature and pH	—	—	—	Gundogdu et al. (2018)
Magnetic HA	DTX	Light and magnetic field/PTT	MDA-MB-231 and NIH/3T3	—	MRI	Zheng et al. (2018b)
Diselenide-containing PMDEGLGAs	DOX	Temperature and redox	—	—	—	Gao and Dong, (2018)
Diselenide PNIPAAm	PTX	Temperature and redox	HepG2	4T1 TBM	—	Xu et al. (2018b)
Poly(PEG-co-PCL)-g-PNIPAAm	DOX	Temperature and reduction	4T1	—	—	Liu et al. (2015)
GC-NBSC CPMs	CPT	Light and pH/PTT	MCF-7	—	—	Meng et al. (2013)
PMPC-b-P(MEMA-hydrazide)	DOX and IR780	Light and pH/PTT	MCF-7/ADR	MCF-7/ADR TBM	FI	Li et al. (2016e)
PEG/PDPA	DOX and Ce6	Light and pH/PTT	MCF-7/ADR	MCF-7/ADR TMB	MRI/FI/PAI	Wang et al. (2016e)
PEG-PU (50 and 100% SS)-PEG	Pyrene	Ultrasound/redox	—	—	—	Tong et al. (2013)
Gold nanorod embedded PEG-b-PHEA-LA-FA	GW627368X	Light and reduction/PTT	SiHa and ME180	S180 TBM	—	Parida et al. (2017)
EC-g-PDMAEMA & EC-g-P(MEO2MA-co-DMAEMA)	DOX	Temperature and CO_2_	—	—	—	Yuan et al. (2016)
PNIPAAm-PBLG CCMs	DOX	Temperature, reduction, and pH	HUVEC	—	—	Qu et al. (2018)
PNIPAAm-S-S-P(αN_3_CL_10_-*g*-PyrePA3/-CholPA7	DOX	Temperature, redox, and ultrasound	HeLa	—	—	Lin et al. (2018)
poly(NIPAM-*co*-SP)	C 102	Light, temperature, and pH	—	—	—	Chen et al. (2015b)
PDMAEMA-*b*-PSPMA	C102	Light, temperature, and pH	—	—	—	Falireas and Vamvakaki, (2018)
PEG-ss-(PDMAEMA-co-PNBM)	NR	Temperature, Light, pH, and redox/PTT	—	—	—	Dong et al. (2018)
(PMAEFc-ONB-PDMAEMA)-x BBAC	NR	Temperature, pH, light, and dual redox/PTT	—	—	—	Zhang et al. (2018a)

P4VP, poly(4-vinylpyridine); PEGS-EVOHS-RA, methoxypolyethylene glycol succinate-succinylated poly(ethylene-*co*-vinyl alcohol)-retinoic acid; poly(diEGMA-*co*-OEGMA300)-*b*-PEHMA, poly[(di(ethylene glycol)methyl ether methacrylate-*co*-poly(ethylene glycol) methyl ether methacrylate 300)-*b*-poly(2-ethylhexyl methacrylate)]; sq GEM, squalenoyl-gemcitabine; PLA, poly(*D,L*-lactide); PLLA, poly(*L*-lactide acid); L35, Pluronic L35; PMEO2 MA, poly(2-(2-methoxyethoxy) ethyl methacrylate); POEGMA, poly(oligo (ethylene glycol) methacrylate); ^131^I-HA, iodine-131-labeled hyaluronic acid; PBMA, poly(*n*-butyl methacrylate); PHMAAm, poly(*N*-hydroxymethylacrylamide); BU-PPG, uracil-based polypropylene glycol; PMPAAm, poly(*N*-(3-methoxypropyl)acrylamide); PPS, poly(propylene sulphide); PNAGA, poly(*N*-acryloylglycinamide); PAN, poly(acrylonitrile); PPEMA, poly(2-(piperidin-1-yl)ethyl methacrylate); PAsp, poly(aspartic acid); IM, imidazole; PSDEA, poly(thiodiethyleneadipate); AIE-1, salicylaldazine; PCPH, poly(6-(4-cyanophenoxy) hexyl methacrylate); AC-CS, acetylated-chondroitin sulfate; Se-based PMs, prepared *via* coupling reactions of PEG; hexamethylene diisocyanate, and bis(hydroxypropyl) selenide; PBLG, poly(benzyl *L*-glutamate); PNBA, poly(o-nitrobenzyl acrylate); HPHEEP-DNQ, hydrophilic hyperbranched polyphosphate-2-diazo-1,2-naphthoquinone; PFMA, poly(furfuryl methacrylate); PC, poly(carbonate); SP, piropyran; EC, ethyl cellulose; PSPMA, poly(spiropyran ether methacrylate); PMA, poly(methacrylate); NP, naphthopyrans; poly(Tyr), poly(α-hydroxy acids); PNHL, poly(ethylene oxide)-*b*-PNIPAAm-*b*-poly(oligolactylmethacryla); a-PXCLs, acetal-poly(4-substituted-ε-caprolactones); CPiPrOx, poly(2-isopropyl-2-oxazoline); PMMA, poly(methyl methacrylate); PDEGMA, poly(ethylene glycol) methyl ether methacrylate); CS, chitosan; ALG, sodium alginate; PNVP, poly(*N*-vinyl-pyrrolidone); PID, poly(N-isopropylacrylamide-*co*-*N,N*-dimethylacrylamide); CSO, chitosan oligosaccharide; PHpr, poly(pseudoamino acid); PHCS, *N*-phthaloylchitosan; PtBA, poly(*tert*-butyl acrylate); PNVIm, poly(*N*-vinylimidazole); POSS, polyhedral oligomeric silsesquioxane; PGMA, poly(glycolmethacrylate); LbL, layer-by-layer films; PDMA, poly[2-(dimethylamino)ethyl methacrylate]; PDEA, poly[(2-(diethylamino)ethyl methacrylate)]; PSS, poly(sodium 4-styrenesulfonate); PMDEGLGA, poly(methoxydiethylene glycol-L-glutamate)s; GC-NBSC, glycol chitosan*-o* nitrobenzyl succinate conjugates; PMPC, poly(methacryloyloxyethyl phosphorylcholine); PMEMA, poly(2-methoxy-2-oxoethyl methacrylate); PDPA, poly(2-diisopropyl methacrylate); PHEA, poly(2-hydroxyethyl acrylate); LA, lipoic acid; CCMs, core cross-linked micelles; PyrePA, pyrenemethyl 4-pentynoate; CholPA, cholesteryl 4-pentynoate; PMAEFc-ONB, poly(2-methacryloyloxyethyl ferrocenecarboxylate)-(5-propargylether-2-nitrobenzyl bromoisobutyrate); and BBAC, *N,N*′-bis(bromoacetyl) cystamine.

### Temperature-triggered polymeric micelles

Temperature is one of the extensively examined stimuli used in DDSs. Throughout heating/cooling operation, temperature alteration prompts the conformational changes of stimuli-responsive PMs to realize temperature-related drug release and intracellular uptake. The structural change of temperature-sensitive PMs induced potential treatment through hyperthermia (Akimoto et al., 2014). The most relevant thermo-responsive co-polymers as drug carriers are PEO–PPO and other polyether amphiphiles, PEO-polyester (e.g., PLA and PCL), and PNIPAAm-based block co-polymers. However, PNIPAAm is extensively studied to engineer thermo-responsive micelles as it displays an LCST of around 32°C near body temperature with sharp phase transition. Above its LCST, PNIPAAm experiences a phase transition from a coiled configuration to a globular configuration. In addition, PHPMA, a highly hydrophilic and biocompatible macromolecule copolymerized with a broad diversity of hydrophobic building blocks, was utilized to produce block co-polymers for the subsequent PMs construction as a substitute for PEG (Sosnik, 2013). Some other LCST-based temperature-responsive polymers as potent drug and/or gene carrier, which have been used so far include, poly(*N*-vinylalkylamide), poly(*N*,*N*-diethylacrylamide), pluronics, tetronics, polysaccharide-, phosphazene, and chitosan-derivatives (Schmaljohann, 2006b).

A new thermo-responsive shell crosslinked nano-system was developed based of PNIPAAm-*co*-poly(*L*-lysine) graft co-polymer that is successfully crosslinked with genipin as a natural crosslinking agent (PNIPAAm-*co*-PLLys)-X genipin (Lai, 2017). MTX was encapsulated on this carrier successfully, and the *in vitro* drug release profile revealed almost complete drug release after 48 h (Tu et al., 2018). Synthesis of two sets of thermo-sensitive four star-armed PMs based on PNIPAAm or PNVCL was reported using bromine terminated pentaerythritol polycaprolactone (PE-PCL-Br) as a macroinitiator through ATRP. The block *co*-polymers (PE-PCL-*b*-PNIPAAm and PE-PCL-*b*-PNVCL) were conjugated with FA and loaded with DOX to provide DOX-FA-PMs. The cellular uptake and MMT assay study demonstrated effective internalization of these PMs into C6 glioma cancer cells with perfect biocompatibility. The *in vivo* assay set expressed high prevention of tumor growth in C6 glioma TBM significantly. The location analysis of DOX in the C6 glioma-induced rat model was accomplished through fluorescent imaging (Panja et al., 2016a). Other thermo-triggered drug delivery PMs systems based on PNIPAAm for tumor targeting are prepared through co-polymerization of NIPAAm with other monomers, for example, acrylamide & *n*-butylmethacrylate (Slaughter and Sunday, 2014) and *N,N*-dimethylacrylamide (DMAAm) & PLA (Yu et al., 2014) for the production of diblock and triblock copolymers, respectively. Furthermore, alg-*g*-PNIPAAm (Ahn et al., 2014) and star β-CD-based PNIPAAm (Song et al., 2016) as temperature-responsive PNIPAAm-based PMs for cancer imaging and therapy are developed.

Synthesis of the thermo-responsive micellar-hydrogel system based on poly(CL-*co*- 1,4,8-trioxa{4.6}spiro-9-undecanone)-*b*-PEG-*b*-poly(CL-*co*-1,4,8-trioxa{4.6}spiro-9-undecanone) (PECT) triblock co-polymer for loading of two therapeutic and diagnostic agents; DOX and ^131^I isotope and their subsequent co-delivery was developed. Temperature-induced self-aggregation of PECT triblock PMs shows synergetic treatment due to the combined chemoradiotherapy. The results confirmed that PECT could be recognized as a potent agent in the co-delivery of DOX and ^131^I, in which the system is diagnosable through fluorescent imaging (Huang et al., 2015).

Block copolymers containing aromatic moieties through polymerization of *N*-(2-benzoyloxypropylmethacrylamide (HPMAm-Bz) or naphthoyl analog (HPMAm-Nt), with *N*-(2-hydroxypropyl) methacrylamide monolactate (HPMAm-Lacy) *via* a PEG-based macro-initiator were synthesized [mPEG-p(HPMAm-Bz/Nt-*co*-HPMAm-Lacy)]. These PMs were loaded with anticancer drugs, PTX and DTX, and their temperature-sensitive release profile was probed. The authors designed these thermosensitive PMs to show that the *π–π* stacking consequence developed by aromatic functionality improves the loading capacity and stability of their DDSs (Shi et al., 2013). Random poly(ether urethanes) PMs were synthesized with tuneable LCST from 30 to 70°C by changing the PEG content. The results demonstrated accelerated DOX release from the nanocarrier above the LCST (Sardon et al., 2015). Preparation of a novel thermosensitive system of poly(*L*-glutamate) (PLG) grafted on 2-(2-methoxyethoxy) ethyl methacrylate (MEO_2_MA) or 2-(2-(2-methoxyethoxy)ethoxy) ethyl methacrylate (MEO_3_MA) *via* click reaction was reported. These “hairy-rod” polypeptides were self-assembled into PMs for loading and releasing of DOX (Ding et al., 2013). Recently, a mini-library of temperature-sensitive six arms star polymers by using xanthate RAFT-agent with hexa functionality and *N*-vinylcaprolactam (NVCL), *N*-vinylpyrrolidone (py), and vinyl acetate (VAc) monomers through RAFT polymerization was developed. Star polymers of PNVCL with variation in homopolymeric arms were created. The aggregation behavior of PMs and their solubility were related to the block order in the arms of star polymers. Interestingly, these libraries of thermo-responsive six arms star polymers displayed dissimilar operations in MTX encapsulation and delivery (Cortez-Lemus and Licea-Claverie, 2017).

### Light-triggered polymeric micelles

UV, visible, NIR, or X-ray as external stimuli cause structural changes in photo-responsive PMs *via* light-induced reactions tailored by light intensity and wavelength and result in the drug release from PMs (Alatorre-Meda et al., 2013). Light responsive PMs are designed by incorporating chromophores within the core or shell of a micelle or at the interface of micelles’ core-corona. The most applied photochromic compounds are azobenzenes and their derivatives, as they can perform a reversible *trans–cis* isomeric transformation. Other reported chromophores in light-sensitive micelles are *o*-nitrobenzyl, coumarin, stilbene, dithienylethene, and DNQ (Movassaghian et al., 2015)*.*

PDT is a favorable treatment based on applying a photosensitizer and light of a particular wavelength for controlling a diversity of tumors. The photosensitizer transforms accessible oxygen to highly ROS in the presence of light irradiation and persuades an intra-tumor cytotoxic response. Hyaluronic acid-*b*-poly(lactide-*co*-glycolide) (PLGA/HA) was synthesized following the loading of PpIX as an effective photosensitizer. PLGA/HA-PpIX micelles have considerable capability for *in vitro* CD44-targeted PDT treatment toward A549 cells (Li X. et al., 2018). Preparation of light-responsive fluorinated polymeric micelle based on perfluorooctanoic acid (PFOC) and branched PEI-loaded photosensitizer Ce6 was developed for PDT and fluorescent imaging. The high efficiency of Ce6-PFOC/PEI in PDT cancer treatment is related to the oxygen-carrying potential of perfluoroalkyl functionalities that supply oxygen and overcome the hypoxia in tumor cells under the conditions of low oxygen content (Wang Q. et al., 2018). A recent report on PMs nanoparticles for PDT treatment is based on the preparation of toluidine blue O-chitosan-poly(propylene sulfide) (TBO-CS-PPS; TCP), following the thymol loading on TCP and subsequent binding to the bacterial biofilm effectively. T-TCP micelles produced ROS by PPS oxidation, triggering thymol delivery, and disrupting biofilm under light irradiation (Wang Z. et al., 2019).

Recently, a system based on light-sensitive micelle plexes NPs was developed for photothermally enhanced delivery. The micelle plexes were produced from poly(amido amine) s modified cholesterol and CXCR4 molecule inhibitor (PAMDCh). NIR dye/IR780 was loaded in cationic PMADCh, which were applied to form IR780@micelle/siRNA polyplexes. Upon laser irradiation, the photothermal effects of IR780 resulted in the disruption of endosomal membranes and facilitated endosomal escape while promoting siRNA transfection (Chen et al., 2018). Synthesis of a set of amphiphilic block co-polymers comprised methyl PEG-*b*-PLys (mPEG-*b*-PLys) with urethane, urea, and thiourea functional groups in their structures to induce hydrogen bonding was disclosed. Subsequent encapsulation of anticancer drug, DOX and photothermal agent, and ICG in PMs provided mPEG-*b*-PLys@DOX-ICG. Constructive effects of strong H-bonding among the inner hydrophobic segment of PMs and at the same time between the hydrophobic parts and drugs include reducing CMCs, increasing micelle stability, improving the drug loading capacity, declining the size of the micelle, and decelerated drug release pattern, respectively. The ICG photothermal effect triggered destabilization of H-bonding and DOX release and considerable enhancement of cytotoxicity under NIR laser irradiation (Li Y. et al., 2018). A novel UV-responsive PMs comprised coumarin ester was announced for killing tumor cells *in vitro* without loading any drugs. (7-diethylaminocoumarin-4-yl)methyl (DEACM) carbonate was chosen as the photo-sensitive group and conjugated with PEG to PEGylated DEACM. Photolysis of DEACM upon UV irradiation can produce carbon dioxide bubbles during micelles degradation. Indeed, *in vitro,* multiple tumor ablation was induced without any anticancer drug through 8-s UV exposure (Zhang et al., 2017).

An innovative oxygen-independent photothermally triggered system combined PTT and PDT was developed by self-assembling PEG-*b*-PCL-*b*-poly(2-(piperidin-1-yl)ethyl methacrylate) (PEG-*b*-PCL-*b*-PPEMA) triblock co-polymer and subsequent *co*-encapsulation with DPAE (diphenylanthracene endoperoxide) and cypate as a fluorescent dye. By NIR irradiation at tumor cells, the PMs induced efficient hyperthermia by cypate through PTT. They triggered large intracellular singlet O_2_ production by DPAE through PDT *via* local photothermal consequence in the interior cores of PMs (Zhou Q. et al., 2018). Another system that applies PTT and PDT combination is a mixed micelle based on the *co*-assembly of PCL-*b*-poly(methoxytri(ethylene glycol) methacrylate-*co*-*N*-(2-methacrylamido)ethyl folate amide) (PCL-*b*-P(TEGMA-*co*-NMFA) and PCL-*b*-poly(diethyleneglycolmonomethylether methacrylate) (PCL-*b*-PDEGMA), which is targeted with folate receptor and encapsulated ICG. Upon NIR irradiation, the toxicity induction by the micelle@ICG has effectively inhibited the growth of HeLa cells (Chien et al., 2018).

### Ultrasound-triggered polymeric micelles

Using localized ultrasound (US) waves as an external physical stimulus for controlling the drug release from ultrasound-sensitive polymer micelles has achieved growing interest due to ultrasound’s safety, inexpensiveness, and non-invasive nature (Awad et al., 2021). In addition, the US is remote management and a non-contact process. This method can be additionally refined by modifying several characteristics containing the properties of the PMs, the interval time of PMs’ exploitation and US utilization, the type of US wave, and the sonication frequency (Entzian and Aigner, 2021).

Five amphiphilic diblock and triblock co-polymers were produced using hydrophilic and hydrophobic oxazoline-based segments. The synthesized PMs were encapsulated with dexamethasone, and their spontaneous and US-mediated release profile was investigated. The results demonstrated that the US intensifies the amount of dexamethasone release by 6%–105% with due attention to the copolymer type, dexamethasone loading on the carrier, and the time, location, and intensity of stimulation (Salgarella et al., 2018).

An engaging system of siRNA micelle-nanobubble (NB) complexes was designed based on gene delivery for tumor therapy. NB was selected as a promising nanocarrier due to the prosperous production of NBs accompanied by its US sensitivity and the potential for passive accumulation in tumor cells. SiRNA micelle-NB was prepared by the interaction of siRNA micelles with a positive charge based on mPEG-b-PLys diblock co-polymer and gas-cored liposomes with a negative charge. The US-mediated siRNA transfection, which gives rise to surpassed therapeutic operation and cancer cell apoptosis, was investigated *in vitro* and *in vivo.* Furthermore, NB diagnosable material in this system has successfully resulted in extravascular ultrasonic imaging (Yin et al., 2013). Later, the co-delivery of siRNA and PTX with this system was reported because of the synergistic result of the two therapeutic agents. Tumor growth was impeded by applying a low amount of PTX in animal models bearing human HepG2 xenograft (Yin et al., 2014).

### Multi-triggered polymeric micelles

Nowadays, designing novel polymeric micelles with the capability of dual or even multiple responsiveness is of great interest. PMs systems with dual responsiveness with at least one physically trigger are dual thermal/light-responsive (Tang et al., 2016; Yang et al., 2019), dual thermal/pH-responsive (Zhang et al., 2007; He et al., 2010; Jia et al., 2010; Lee et al., 2010; Yang et al., 2010; Jain et al., 2011; Ding et al., 2013; Wu et al., 2014; Khine et al., 2015; Chen et al., 2017c; Zhou Z. et al., 2018; Ghamkhari et al., 2018; Uz et al., 2019), dual light/magnet-responsive (Zheng S. et al., 2018), dual thermal/redox-responsive (Liu et al., 2015; Xu J. W. et al., 2018; Gao and Dong, 2018), dual light/pH-responsive (Meng et al., 2013; Li Z. et al., 2016; Wang T. et al., 2016), dual ultrasound/reduction-responsive (Tong et al., 2013), dual light/reduction (Parida et al., 2017), and dual thermal/CO_2_-responsive (Yuan et al., 2016). Triple-triggered PMs are thermal/reduction/pH-responsive (Sun et al., 2018b; Qu et al., 2018), thermal/ultrasound/reduction-responsive (Lin et al., 2018), and light/temperature/pH- responsive (Falireas and Vamvakaki, 2018). Even quadruple temperature, pH, redox, and UV light (Dong et al., 2018), quintuple light, temperature, pH, and dual redox stimuli-responsive PMs (Zhang K. et al., 2018) are synthesized and applied in the controlled delivery of therapeutic cargos.

Systems with dual photo-responsive and temperature-responsive characteristics are created by adding chromophore functional groups to the thermo-responsive systems (Belmonte et al., 2020). Recently, the dual thermal/light-responsive PMs by the preparation of random and block co-polymers comprised light-sensitive spiropyran, and oligo (ethylene glycol) methylethermethacrylate was reported. UV light irradiation and heating of the sample stimulated increased drug release (Yang et al., 2019).

Combining pH-responsive co-polymer with a photosensitizer was established to overcome the drug resistance in targeted tumor therapy through chemo-PTT. Light-sensitive *o*-nitrobenzyl succinate (NBSC) that was grafted onto glycol chitosan (GC) and then subsequent crosslinking with glutaraldehyde (GA) lead to the formation of the dual light/pH-responsive-crosslinked PMs (CPMs). Fast release of CPT was observed during light irradiation at low pH (Meng et al., 2013). A multitasking micelle for combinational photo/chemotherapy displayed MR, fluorescence, and PA multimodal tumor imaging was developed. The micelle was composed of a diblock copolymer PEG-*b*-poly (tertbutylmethylacrylate-*co*-hydroxylmethacrylate), photosensitizer Ce6, Gd^3+^ as MR T1-weighted MR agent, and prodrug DOX. Upon NIR laser irradiation, the micelle induced ROS creation and local heat production for PDT and PTT and diagnosed *via* PAI. In addition, the micelle could construct a magnetic resonance signal at an acidic medium to implement MRI (Wang T. et al., 2016).

Among dual responsive PMs, thermal/pH-responsive is the most studied one. The most recent report is the dual delivery nanoscale device (DDND) based on a pentablock copolymer system composed of amphiphilic pentablock copolymers based on Pluronic ^®^ F127 copolymers and various amine-containing methacrylate monomers for combined delivery of microRNA (miR-345) and GEM (Uz et al., 2019).

Dual thermal/pH-responsive supramolecular micelles were designed from star polymer β-CD-PNIPAAm and benzimidazole terminated PCL (β-CD-PNIPAAm and BM-PCL). The delivery of DOX from supramolecular PMs was accelerated at low pH at 37°C (Zhou Z. et al., 2018). Another thermal/pH-responsive PMs was introduced based on four-armed star-like PMs that were prepared from hyperbranched aliphatic polyesters (HAPs)-*g*-PLC-*b*-PNIPAAm block co-polymer. The first block (PCL) is prepared *via* ROP, and the chain extension with the second block (PNIPAAm) is performed *via* RAFT polymerization. DTX was loaded on these biodegradable PMs with high encapsulation efficiency, and the release rate was controlled *via* pH and temperature (Ghamkhari et al., 2018).

A creative quadruple responsive copolymer based on PEG-ss-poly (DMAEMA)-co-poly (2-nitrobenzyl methacrylate) [PEG-ss-(PDMAEMA-co-PNBM)] comprising disulfides unit was developed. Self-assembling this copolymer generates PMs with hydrophobic PDMAEMA-*co*-PNBM core and hydrophilic PEG coronas that are multi-responsive to temperature, light (UV), pH, and reduction (*via* the presence of dithiothreitol, DTT). The *in vitro* release profile was investigated by the use of hydrophobic NR drug. Each stimulus has a specific effect on these multi-responsive PMs; as the temperature changed the size of the micelle, the micelle swelled in low pH, a few amounts of DTT disarranged the micelle configuration, and irradiation of UV light caused the dissolution of the micelle construction (Dong et al., 2018).

## Nanogels

The term “nanogels” (NanoGel™) is used to define nanosized particles (10–100 nm) formed by crosslinked polymer networks, which are able to swell in a suitable solvent and absorb large quantities of water (Molina et al., 2015; Shah et al., 2020).

Crosslinks are essential for nanohydrogel structural stability because they prevent polymer chain dissolution in the aqueous environment. NGs, also called hydrogel nanoparticles, are found in a wide variety of applications in biomedical fields, such as DDS and bioimaging (Sasaki and Akiyoshi, 2010; Lu et al., 2010; Mauri et al., 2021).

The recent development of different preparation techniques made it possible to regulate the essential final parameters such as size, shape, and yield. NGs are prepared by various methods of co-polymerization *via* reaction among hydrophilic monomers and difunctional cross-linkers or physical cross-linking agents (Mauri et al., 2021).

Traditional uncontrolled free-radical polymerization in the presence of a cross-linker combines the two processes of polymerization and crosslinking in one reaction (Neamtu et al., 2017). NGs have emerged as a platform to encapsulate versatile therapeutic agents within their networks to be applied for therapeutic applications (Soni et al., 2016b; Saracoglu and Ozmen, 2021). This section comprehensively discusses external stimuli-responsive NGs and their current applications in biomedical fields.

### Nanogels in therapy and diagnosis

The most important NGs’ features are ease of preparation, biocompatibility, degradability, swelling in aqueous media, the high adsorption capacity of therapeutic agents, small particle sizes, electromobility, and colloidal stability (Ghasemiyeh and Mohammadi-Samani, 2019).

NGs are good carriers for DDS due to their specific properties (Soni et al., 2016b; Mohammadi M. et al., 2020):

• The small particle size and easy surface manipulation inhibit rapid phagocytic cell clearance and promote drug targeting by passive and active strategies.
• Controlled and sustained drug release properties at target sites enabled them to promote activity tasks and reduce other side effects.
• High drug loading potent without chemical reactions is suitable to preserve drug activity.
• Tiny particles’ volume allows them to penetrate into a particular tissue (e.g., tumor site) through the paracellular or transcellular passages (Gonçalves et al., 2010).
• Both types of drugs (hydrophilic and hydrophobic) could be entrapped in NG networks.

However, using expensive techniques for preparing some NGs and removing solvents and surfactants from final products are disadvantages of such systems (Zhou et al., 2020).

NGs can be classified as non-responsive and stimuli-responsive. Non-responsive NGs swell consequently to the water absorption. In contrast, responsive NGs can swell/deswell after exposure to environmental changes (such as temperature, pH, light, electric field, ultrasound, or magnetic field (Lim et al., 2014).

As a powerful nanoscale candidate for DDSs, the capability of NGs for loading and carrying medications has been explored by many researchers (Soni et al., 2016a).

**FIGURE 3:**
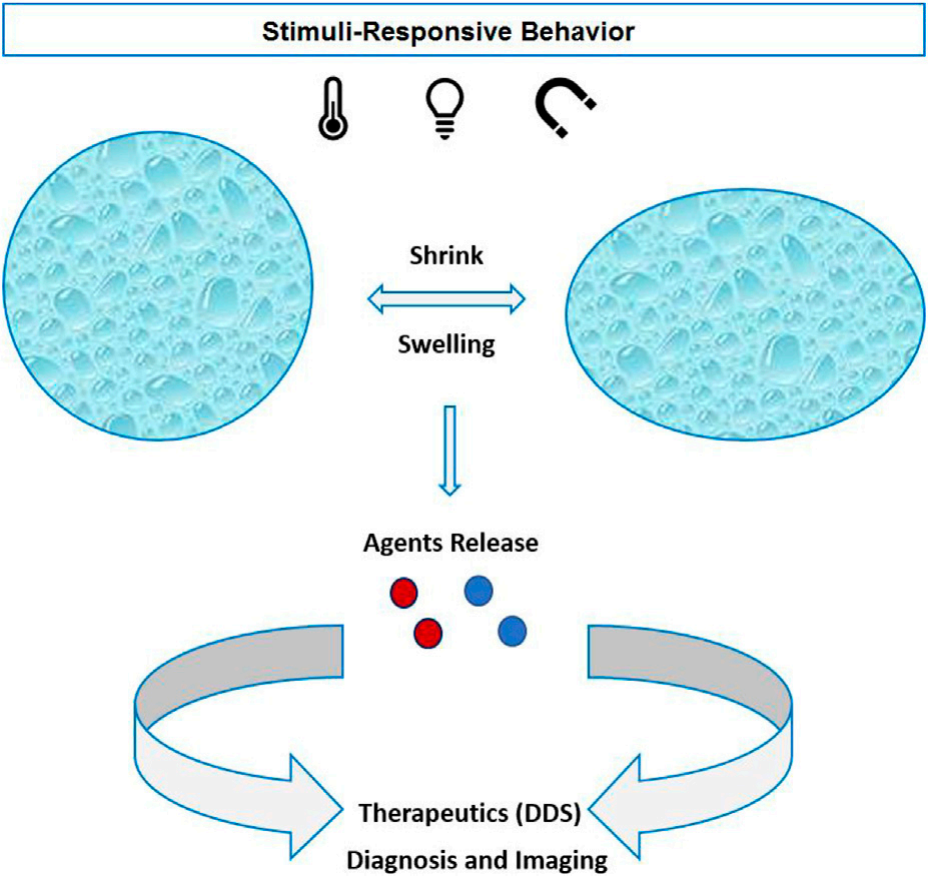
Physical stimuli-responsive nanohydrogels with shrink/swelling behavior and active agents released for therapeutics or diagnostic purposes.

According to the chemical composition of the stimulus-responsive NGs, they begin to swell or shrink followed by releasing the cargo as the stimulus is switched “ON.” Therefore, the external stimulus can control the release profile of the therapeutic agents locally and yield a higher effective treatment (Figure 3). NGs are also used as a carrier for imaging probes and contrast agents. Small molecules based on gadolinium (Gd) and manganese (Mn) are introduced in nanohydrogels to induce contrast in MRI (Soleimani et al., 2013; Gallo et al., 2020). Lux et al. (2015) developed a copper-64 cross-linked NG for PET radiotracers of tumors and metastases. Furthermore, hybrid systems based on nanohydrogels and inorganic NPs (quantum dots and AuNPs) are being developed for optical imaging purposes (Molina et al., 2015).

Currently, just a few NGs formulations for subcutaneous delivery have reached clinical trials (Kitano et al., 2006; Kageyama et al., 2013; Saito et al., 2014; Jiang L. et al., 2020) because some parameters have to be optimized, such as rapid spleen clearance, the surface charge that influence opsonization, degradation kinetics, and burst drug releases (Soni et al., 2016b).

This section reviews the NG-based drug carriers responding to physical factors. Table 2 summarizes recent studies in this field.

**TABLE 2:** Physically triggered NGs concerning the type of stimulus and cargo for *in vitro* and *in vivo* investigation, therapy, and diagnosis.

NG structure	Cargo	Physical stimulus/therapy	*In vitro* assay	*In vivo* assay	Imaging modality	Reference
Spermine-modified PNIPAAm	Cis-Pt	Temperature	HT-29	—	—	Ghasemi et al. (2022)
Lysine-modified PVCL-*st*-PEG	DOX	Temperature	MCF-7	—	—	Farjadian et al. (2019b)
PNIPAAm	Lopinavir	Temperature and redox	—	—	—	Town et al. (2019)
p(NIPAAm-co-NHMAAm-co-SCC)	5-FU SCC	Light and Temperature/PTT	L929	—	—	Chang and Tsai, (2018)
NIPAAM/NPAM/NAPr-MBA-A-Pro-OH	Nile Blue A	Temperature	—	—	—	Salinas et al. (2018)
(PNIPAAm-co-PDEMAEMA)/SA-MGO	DOX	pH, temperature, and magnetic field	MCF7	—	—	Bardajee and Hooshyar, (2018)
Hep-F127	Cis-Pt and curcumin	Temperature and pH	—	Mus musculus var. albino mice	—	Nguyen et al. (2018)
Alg-CD	5-FU	Pressure	HT-29	—	—	Hosseinifar et al. (2018)
CS-g-PNIPAAm	Levofloxacin	Temperature	—	—	—	Shin et al. (2018)
PDMAEMA	DOX	Temperature and pH/radiotherapy	4T1	4T1 tumor-bearing BALB/c mice	Imaging using gamma camera	Maiti et al. (2018)
	^131^I labeled albumin					
CS-g-PNIPAAm	Curcumin	Temperature	NIH-3T3	—	—	Luckanagul et al. (2018)
			HeLa			
PEI-PNIPAAM-PEI	DOX	Ultrasound and redox	HEK293	—	—	Shen et al. (2018)
			Huh7			
dPG-co-PNIPAAm	Etanercept	Temperature	—	—	—	Giulbudagian et al. (2018)
dPG-co-PNIPAAm	Indodicarbocyanine	Temperature	—	—	—	Jung et al. (2018)
PEGMEMA-co-PFuMaMA-co-PHEMA	DOX and Cy5	Temperature and pH	MDA-MB231	—	FI	Chambre et al. (2018)
			L929			
SP-MA	DOX	UV light, temperature, and redox	MCF7	—	—	Chen et al. (2017a)
PNIPAAM-co-PAA	Methylene blue	Temperature and pH	—	—	—	Pruettiphap et al. (2017)
*β*-CD/PNIPAAM-PAA	DOX	Temperature and pH	KB	—	—	Yi et al. (2017)
Protamine/PAA-b-PNIPAAm	DOX	Temperature, pH, and enzyme/PDT	MCF-7 MCF-7/ADR	—	FI	Don et al. (2017)
	5-FU and RB					
CS-g-PNVCL	NIPAAm	Temperature and pH	L929	—	—	Indulekha et al. (2017)
			NIH 3T3			
SMGO/P(NIPAAM-co-AA)	DOX	Temperature and pH	HeLa	—	—	Bardajee et al. (2017)
Pluronic F127	Lidocaine	Temperature	—	White rabbit	—	Sharma et al. (2017)
	Prilocaine			Wistar rat		
PNIPAAm/CS/MWCNT	DOX	Temperature and pH	—	—	—	Luo et al. (2017)
PNIPAAM- dPG	DOX	Temperature	HeLa	Nude mice	—	Molina et al. (2017)
PE-PCL-b-PAA	DOX	Light	C6	C6 tumor-bearing Sprague–Dawley rat	—	Panja et al. (2016b)
NOCS-g- poly(NIPAAm-IA-AMPS)	DOX	Temperature and pH	MCF-7	—	—	Verma et al. (2016)
			MDA-MB231			
			MCF10A			
PNIPAAm-co-PMAA-co-PHEMA)	Cis-Pt	Temperature and pH	—	—	—	Kurd et al. (2016)
PAMAM G3–PNIPAAm	5-FU	Temperature	MCF7	—	—	Le et al. (2016)
PNIPAAm	Donepezil	Temperature	—	Zebra fish	—	Kalaiarasi et al. (2016)
PEO-PPO-PEO	Muscone	Temperature	—	Male New Zealand albino rabbits	—	Wang et al. (2016b)
PNVCL-co-N-succinimidyl methacrylate	DOX	Temperature and redox	—	—	—	Peng et al. (2016)
HPMC	Insulin	Temperature and pH	—	—	—	Zhao et al. (2016)
mPEG-PLGA-BOX	Bevacizumab	Temperature	RF6A	—	—	Hu et al. (2015a)
PPEGMA-co-PHPMA-co-PADMA-PAMAM-CD	DOX and ICG	Light/PTT	HepG2	CD-1 (ICR) mice	FI	Zan et al. (2015)
CS-g-PNIPAAm	Hydroxyl-CMP	Temperature	L02	—	—	Wang et al. (2015)
PNVCL-co-PDMAEMA	5-FU	Temperature and pH	—	—	—	Sudhakar et al. (2015)
PEG-co-EGDMA-co-DMAEMA	p-100 peptide KVPRNQDW	Iontophoresis	—	B16-F1 cell-bearing mice	—	Toyoda et al. (2015)
PNIPAAm-PMAA-PPy	Ciprofloxacin	Temperature and pH	*Pseudomonas aeruginosa*	White rabbits	—	Davaran et al. (2015)
dPG-PNIPAAm	Transglutaminase 1 protein	Temperature	—	—	—	Witting et al. (2015)
P(LAEMA)-b-P(DEGMA-st-MBAm)/galactosylated	Iodoazomycin arabinofuranoside	Temperature	HepG2	—	—	Quan et al. (2015)
mPEG-IS	PTX	Temperature and pH	—	BALB/c mice	—	Chen et al. (2014)
PNVCL-co-PDMAEMA	Rh B	Ultrasound, Temperature, and pH	—	—	—	Demirel and von Klitzing, (2013)
mPEGMA-co-PNIPAAm-co-PMAA-st-MBAM	Cis-Pt	Temperature and pH	—	Balb/C mice were	—	Peng et al. (2013)
P(NVCL-co-AGA)	5-FU	Temperature and pH	—	—	—	Madhusudana Rao et al. (2013)
PNIPAAM-co-PAA	DOX	Temperature and pH	HepG2	—	—	Xiong et al. (2011)

p(NIPAAm-co-NHMAAm-co-SCC), poly(NIPAAm-co-N-(hydroxymethyl)acrylamide-co-sodium copper chlorophyllin); (NIPAAM/NPAM/NAPr)-MBA-A-Pro-OH, (NIPAAM/NPAM/N-acryloylpyrrolidine)-N,N′-methylenebis(acrylamide)- N-acryloyl-L-proline; SAlg-MGO, sodium alginate-magnetic graphene oxide; Hep-F127, heparin-Pluronic F127; PEGMEMA-co-PFuMaMA, PEGmetacrylate-co- furan-protected maleimide-containing methacrylate; SP-MA, 3′-dimethyl-6-nitro-spiro(2H-1-benzo-pyran-2,2′-indoline)-1′-(2-methacryloxyethyl); protamine/PAA-*b*-PNIPAAm, protamine/poly(acrylic acid)-*b*-PNIPAAm; SMGO/P(NIPAAM-co-AA), salep modified graphene oxide/poly(NIPAAM-co-AA); MWCNT, multiwalled carbon nanotubes; dPG, dendritic polyglycerol; PE, pentaerythritol; NOCS-g-poly(NIPAAm-IA-AMPS), N,O-carboxymethyl chitosan-g-poly(NIPAAm-co-1-propene-2-3-dicarboxylate-co-2-acrylamido-2-methyl-1-propanesulfonate); PAMAM G3–PNIPAAm, polyamidoamine dendrimer (G3.0)-PNIPAAm; HPMC, hydroxypropyl methylcellulose; PLGA-BOX, poly(lactic-co-glycolic acid)- 2,2-bis(2-oxazoline); PPEGMA-co-PHPMA-co-PADMA-PAMAM-CD, poly[PEG monomethyl ether metharcylate]-co-poly(N-(2-hydroxypropyl)methacrylamide)-co-poly(N-adamantan-1-yl-2-methacrylamide)-PAMAM-CD; P(LAEMA)-b-P(DEGMA-st-MBAm), P(lactobionamidoethyl methacrylamide)-b-P(di(ethylene glycol)methylethyl methacrylate-crosslinked- N,N′-methylenebisacrylamide); mPEG-IS, mPEG2000-isopropylideneglycerol; and AGA, acrylamidoglycolic acid.

### Thermo-responsive nanogels

Thermo-responsive NGs can be classified into two main groups (Hosseini et al., 2016; González-Ayón et al., 2020). In one type of such NGs, the size increment could be occurred by elevating the temperature; in the other one, the NGs are shrunk beyond the volume phase transition temperature (VPTT). As a remarkable precursor for synthesizing thermal responsive compositions, PNIPAAm is in the spotlight of such studies due to the LCST (i.e., 32°C) close to the human body temperature (Priya James et al., 2014). Exploiting the dependency of PNIPAAm phase transition to the temperature tolerance, many NG-based DDSs have been developed and extensively investigated for drug-loading cytotoxicity and biodegradability assessment and temperature-dependent releasing profile (Le et al., 2016; Luo et al., 2017; Bardajee and Hooshyar, 2018; Yang Q. et al., 2018; Chang and Tsai, 2018; Town et al., 2019).

As a theranostic multi-responsive NGs, PDMAEMA was synthesized to load chemotherapy drug (DOX) and radioisotope (131I) labeled albumin simultaneously. The NGs showed a proper responsivity to temperature and pH so as it was in a solution form at room temperature while transformed into the gel near body physiological temperature. Moreover, the drug release accelerated in tumor cells due to pH effect. Surprisingly, the nanocarrier exhibited a biodegradable manner at 37°C and pH of 5.8. Furthermore, MTT assay on 4T1 cells revealed that it could be biocompatible and cause significant growth inhibition by sustained drug release. Administration of PDMAEMA gel @^131^I-BSA/DOX in BALB/c mice with 4T1-induced murine breast cancer showed excellent tumor growth inhibition among the other groups. In addition, gamma imaging showed remarkable retention of ^131^I-BSA in the tumor about 48 h after injection (Maiti et al., 2018).

Recently, HIV drug lopinavir was loaded in a polymeric NG (PNIPAAm) prepared in different sizes (Town et al., 2019). The injectable NG disclosed a dual responsive behavior with changes in temperature and ionic strength, which promise it as an intelligent carrier with a high potential of loading and releasing the drug. A multi-responsive NG was designed to achieve more effective drug release by copolymerizing PNIPAAm and PDMAEMA containing sodium alginate and magnetic GO. Exposure to various temperatures and pH and the presence of a magnetic field affected the DOX release rate. The cytotoxicity test on MCF-7 cells indicated desirable biocompatibility for the synthesized NG (Bardajee and Hooshyar, 2018). Another nano-platform conjugated with NOCC was developed so that the DOX release was dependent on temperature. The viability tests unveiled that the MCF-7 and MDA-MB231 tumor cells are more killed than MCF10A normal cells.

Moreover, they observed cell cycle arrest triggering apoptosis death in MCF-7 cells by the nano-carriers (Verma et al., 2016). Poly (NIPAAM-MAA-VP) was used as a nanocarrier with dual responsiveness to thermal/pH stimulation to release ciprofloxacin’s antibacterial agent. It showed a substantial result in animal models (Davaran et al., 2015).

**FIGURE 4:**
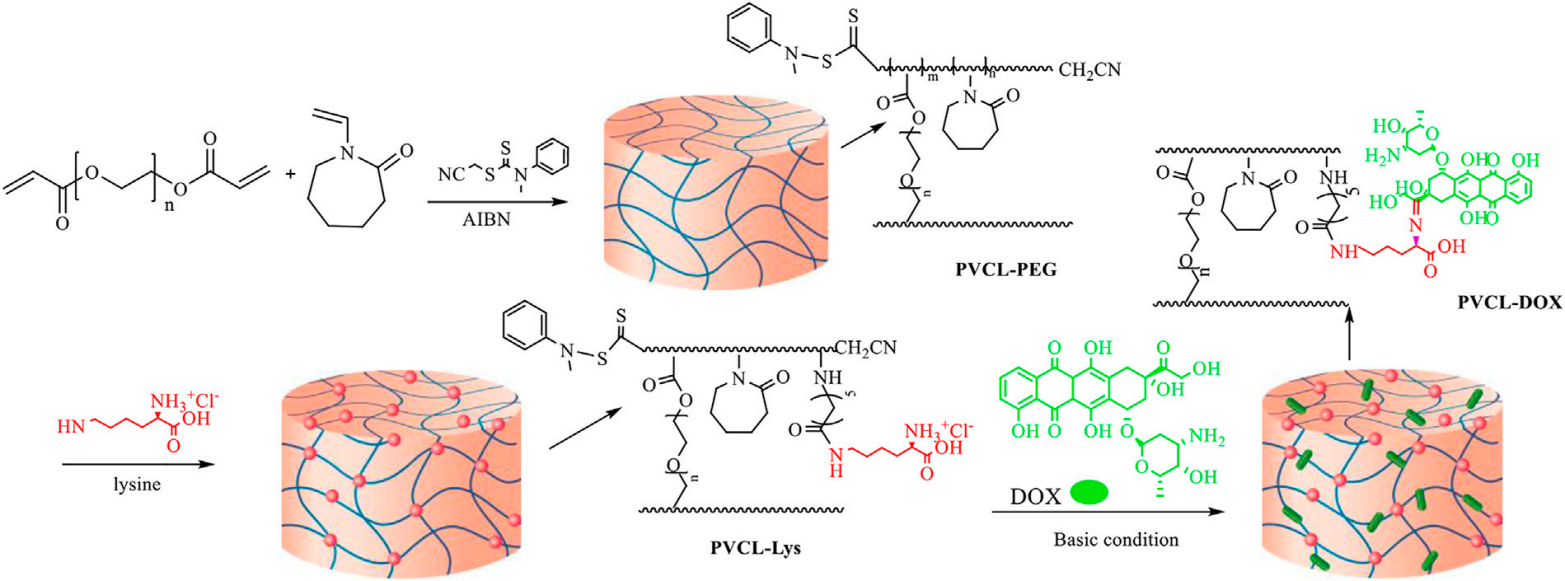
Synthetic strategy in forming PVCL-DOX NG (Farjadian et al., 2019b). No permission is required.

Thermo- and pH-responsive NG delivery system based on lysine modified-polyvinylcaprlactam (PVCL) conjugated with DOX was established and showed high uptake in the MCF-7 cell line. The synthetic strategy depicted in Figure 4 was based on RAFT through copolymerization of PVCL with PEG diacrylate to form PVCL–PEG. Then, PVCL–PEG was modified with L-lysine aminoacid to form PVCL-Lys, which has thermo sensitivity around 38°C and an amino-free site for conjugation of DOX (Farjadian et al., 2019b). Finally, in a pH-responsive linkage, Schiff base reaction, DOX was linked to NG and formed PVCL-DOX.

### Light-responsive nanogels

Taking advantage of light penetrating the materials, the light-responsive DDSs have become the spotlight of many studies in the field of drug delivery (Jiang Z. et al., 2020). For instance, PPEGMA-*co*-PHPMA-co-PADMA-PAMAM-CD NG was fabricated to load with ICG and DOX, exhibiting strong NIR-light sensitivity. Using PTT and chemotherapy simultaneously leads to effective results in *in vitro* and *in vivo* assays (Zan et al., 2015). In another research, laser light-triggered DOX release from PE-PCL-*b*-PAA NG was studied. The NG has a significant destructive effect on the proliferation of C6 glioma cancer cells and an inhibition effect on tumor growth of C6 tumor-bearing Sprague–Dawley rats (Panja et al., 2016b). Recently, by merging the synergistic effect of hyperthermia and drug release from a thermo-sensitive NG, a notable cell killing was achieved. The p(NIPAAm-*co*-NHMAAm-*co*-SCC) NG was loaded with 5-FU and sodium copper chlorophyllin (SCC), which produces heat exposure to green light (Chang and Tsai, 2018). Isomerization of hydrophobic spiropyran to hydrophilic merocyanine due to UV light exposure caused swelling up the SP-MA NG. In addition, the authors claimed that MCF-7 cell killing increases after treatment with UV-irradiated DOX-loaded NG (Chen S. et al., 2017). In a study combination of GO as a photothermal agent, DOX and HA led to a novel NG, making it a multi-purpose carrier. The elevation of temperature due to absorbing NIR light by GO can enhance DOX release from NG. The platform showed acceptable biocompatibility on MDCK cells, while an effective killing trend on the A549 cell line (Khatun et al., 2015).

A novel type of transdermal microneedle arrays made of alginate hydrogel with PLA and the peptide-nucleic acid coating was applied for mRNA sampling from skin interstitial fluid. Microneedles were removed from skins, and in a UV-triggered release process, the adsorbed mRNA was released and detected. This technology enables the detection of mRNA-based biomarkers and could be recognized as a specific tool in personalized medicine (Al Sulaiman et al., 2019). A light-responsive transdermal delivery system based on PHEMA and PEG dimethacrylate nanohydrogel was also applied for Ibu delivery (Hardy et al., 2016).

### Other stimuli-responsive nanogels

Owing to the interesting properties of ultrasonic waves like bio-safety, penetration in soft tissue and controllability, it has been utilized as a powerful tool for stimulating DDSs. Loading of perfluorohexane (PFH) on PEI-PNIPAAM-PEI NG resulted in an ultrasound-responsive structure that releases DOX immediately after cavitation induced by ultrasonic waves. The DOX-loaded NG showed comparable cytotoxicity on HEK293 and Huh7 cell lines compared to free DOX. It also demonstrated more cell growth inhibition in tumor cells than in normal ones (Shen et al., 2018).

Improving wound healing and increased therapeutic gain for frostbite were reported after treatment of rats’ skin with GLT NG containing triterpenoids drug in combination with ultrasound waves (Shen et al., 2016). Pressure as another physical tool to stimulate nano-hydrogel to release anticancer drug (5-FU) was used on Alg-CD nano-carriers, in which the mortality rate of colon cancer cells (HT-29) was elevated for 5-FU-loaded Alg-CD NG in comparison with free 5-FU (Hosseinifar et al., 2018). Iontophoresis using a small electric current has proven as a non-invasive method to enhance transdermal drug delivery. To overcome the low accumulation of antigens in the epidermis, NGs containing gp-100 peptide KVPRNQDWL were developed. The anticancer effect of the NG was explored on B16-F1 cell-bearing mice in combination with iontophoresis where the tumor growth was significantly suppressed by the treatment method (Toyoda et al., 2015).

## Liposomes for delivery of therapeutic and imaging agents

Among the other nanostructures to deliver the cargo to the desired site, liposomes are one of the first and most investigated nanocarriers due to their biocompatibility, low side effects or cytotoxicity, ease in biodegradation under physiological conditions, and desired properties in loading and delivery of the entrapped cargo. The spherical shape of liposomes formed by the bilayer of lipids allows them to mimic the cell membrane and load both hydrophobic and hydrophilic drugs (Bangham and Horne, 1964). Due to the different extravasation behavior of various types of tumors, the passive release of the drug by liposomes proved a low performance, and many studies have extensively investigated the potential of liposomes to respond to physical stimulus sources to release the drugs in a time- and location-dependent manner (Ta and Porter, 2013). Once liposomes carrying drugs accumulate at the desired site, external sources like hyperthermia or light generators focusing on the site can trigger the drug release process *via* the disordering of lipid shells of the liposomes.

### Thermo-responsive liposomes

Using particular physical sources to increase the temperature above the physiological condition but not more than 42°C converted mild hyperthermia into a powerful tool for targeting drug delivery purposes. As the sources can act selectively in ON/OFF modes during a predefined interval and irradiate in a user-adjusted location, they have shown promising results in combination with chemotherapy agents (Franckena et al., 2007). Hyperthermia would enhance the therapeutic effect by the increase in blood flow and expansion of vascular pores to improve the extravasation of liposomes into the tumor as well as by exploiting the thermo-responsive property of liposomes to release the drugs more effectively (Huang et al., 1994; Kong et al., 2000b). According to the literature, thermo-sensitive liposomes are classified into three main categories: 1) traditional thermo-sensitive liposomes (TTSL), 2) lysolipid-containing thermos-sensitive liposomes (LTSL), and 3) polymer-modified thermo-sensitive liposomes (PTSL).

**FIGURE 5:**
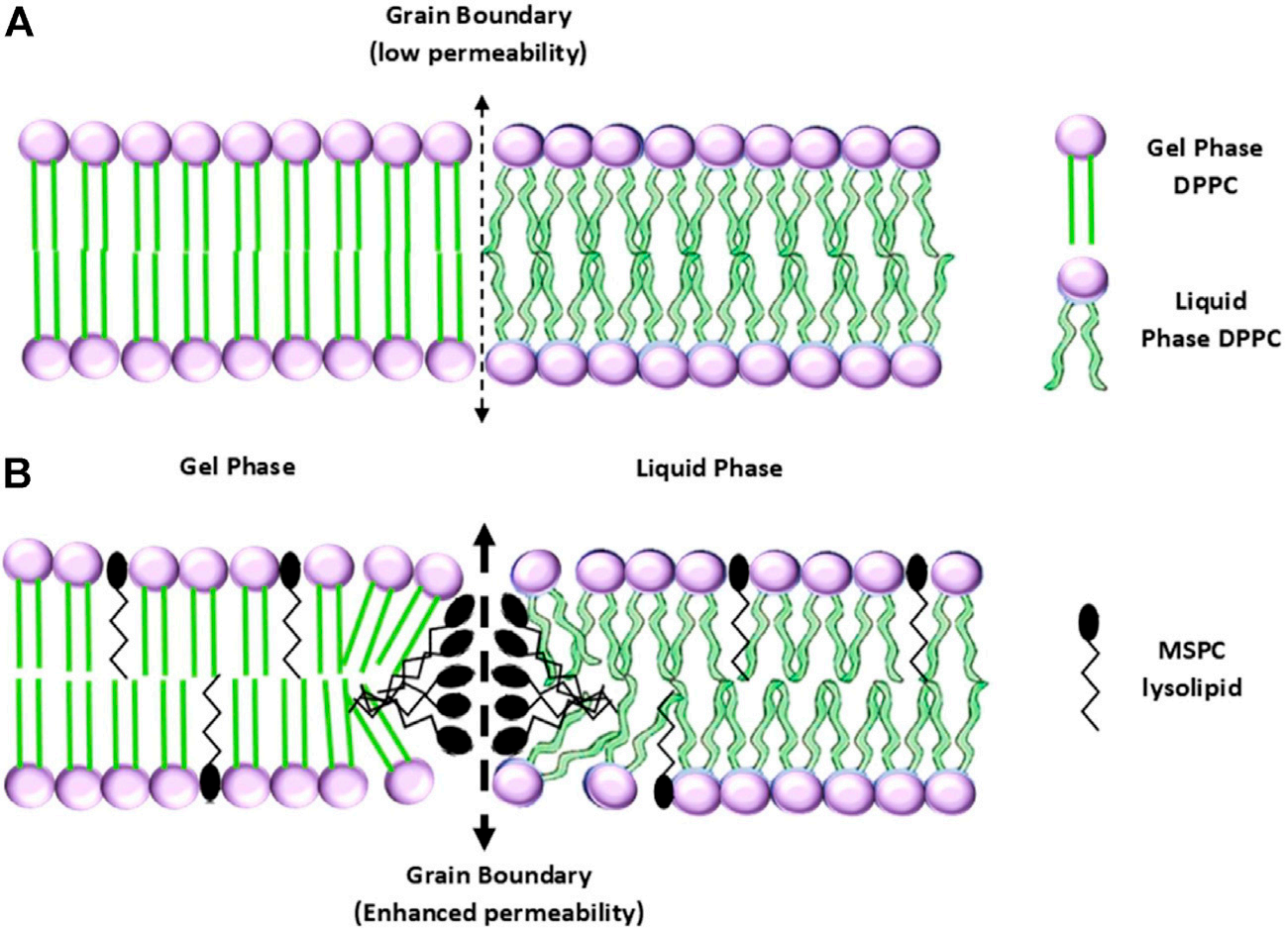
**(A)** Configurations of a TTSL (up) and an LTSL (down) before, and **(B)** after the transition from gel to liquid phase (Abuwatfa et al., 2022). No permission is required.

Considering the phase transition from gel to liquid, Yatvin et al. (1978) developed the first version of TTSLs, in which the liposome starts to melt at the transition temperature (T_c_) while experiencing heating (Figure 5). Although they showed the great potential of liposomes to respond to hyperthermia, they owned a low amount and rate of neomycin release. The issue was addressed by introducing the other lipids to the liposome structure, which leads a 100 times greater release rate at 44°C than 37°C (Bassett et al., 1986; Gaber et al., 1995). In another study, it has been shown that dFdC (pyrimidine analog Gemcitabine) is effectively released from the liposome at 43°C compared to 37°C (80% vs. 20%) (Limmer et al., 2014).

As for *in vitro* and *in vivo* studies, the vinorelbine-containing liposomes (Thermo-Vin) were designed and investigated at 37°C or 42°C (using an RF generator) on H22 cancer cells. The *in-vitro* study exhibited that both Free-Vin and Thermo-Vin groups significantly decreased the H22 cell viability after 24 h. Moreover, the accumulation of the drug into the tumor was 15 times greater for the Thermo-Vin+RF group than the Free-Vin one. The Kaplan–Meier analysis showed that the Thermo-Vin+RF group owned the longest mice survival among all groups (Wang S. et al., 2016). Another study investigating the capability of TTSLs to carry dual drugs of DOX and vincristine proved that the liposomes released around 85% of both cargos under 42°C temperature only in the first 5 min, while at 37°C the amount was less than 10% even for 30 min heating (Li M. et al., 2016). Another thermo-sensitive liposome carrying DTX (DTX-TL) showed a release amount of 40 and 15% for 42 and 37°C, respectively. The tumor growth analysis unveiled that the human breast tumor-bearing mice treated with DTX-TL had the greatest inhibitory impact on the tumors (Zhang H. et al., 2014).

As discussed in many studies, the TTSLs are stimulated under long-time exposure to high temperatures, in which healthy tissue necrosis may occur. To overcome the shortage of high thermal doses by TTSLs, the concept of incorporating lysolipids or thermo-sensitive polymers into the liposomes was proposed. In this regard, the modified liposomes release the drug under mild hyperthermia (39–42°C) in a burst manner, allowing the liposomes to be more clinically applicable (Abuwatfa et al., 2022).

The idea of modifying thermo-responsive liposomes using lipids with single-cyl chains-lysolipids dates to 1999 by Anyarambhatla and Needham (1999). The lysolipids give rise to a lower T_c_ while triggering a rapid release of the drug due to their accumulation at the boundary and making stable pores (Figure 5) (Landon et al., 2011). For example, the incorporation of a lysolipid into liposomes decreased the T_c_ from 43 to 40°C, while a half amount of the drug was rapidly released after 20 s of heating. In addition to taking advantage of falling below the necrosis threshold, the LTSL exhibited a promising *in vivo* result in prohibiting tumor growth (Needham et al., 2000). In another study, it has been reported that LTSL-containing DOX in combination with hyperthermia has a significant therapeutic impact on the FaDu human tumor-bearing mice. The highest accumulation of DOX in the tumor was attributed to the group treated with LTSL under 42°C heating (Kong et al., 2000a). At the clinical trial level, ThermoDOX is the only thermo-sensitive liposomal product based on lysolipid, which underwent clinical trials (HEAT trials) (Bulbake et al., 2017). At this time, the ThermoDOX is used in ongoing clinical trials in combination with focused ultrasound (PanDOX: NCT04852367) and magnetic resonance-guided high-intensity focused ultrasound (HIFU) (de Maar et al., 2020).

Since, in the biological environment, LTSL suffers the gradual desorption of lysolipids from the liposome shell (Ta et al., 2010; de Smet et al., 2011), incorporating thermos-responsive polymers into the liposomes is another approach to bring transition temperature below the thermal necrosis threshold (Bi et al., 2019). Once the thermo-sensitive polymer experiences a temperature over LCST, it onsets to shrinkage and dehydration, which causes disorder in the integrity of the liposome shell. It releases the entrapped drug. As an early work, hydrophobic PNIPAAm was integrated into the liposome structure by Ringsdorf et al. (1991). For another study, modification of DPPC liposomes with p(NIPAAm-co-AAm) led to a synergistic effect in the release of DOX at 40°C compared to the unmodified version (65% vs. 40% drug release). The combination of NIPAAm and AAc followed by incorporation into the liposome yielded a PTSL that released 65% DOX within 5 min of hyperthermia exposure (39°C). In comparison, at physiological temperature, the PTSL prevents around 90% of the drug from being released (Han et al., 2006). In addition, the impact of a DOX-loaded PTSL in combination with MR-guided focused ultrasound was investigated *in vitro* on MCF-7 breast cancer cells and *in vivo* on tumor-bearing Fischer rats. The study showed a great potential of the PTSL as a thermo-sensitive drug carrier (Ta et al., 2014). In a recent study, it has been proven that P(NIPAAm-DMAAm)-DSPE integrated into liposomes released a higher amount of PTX at 40°C compared to unmodified liposomes. The PTSL showed low cytotoxicity while carrying the drug. They reduced the viability of A549 lung cancer cells (Xi et al., 2020).

### Light- and ultrasound-responsive liposomes

The other physical stimuli sources, rather than hyperthermia generators, aid the liposomal nanocarriers to release their content effectively inside the target on-demand without inducing intense side effects like thermal necrosis. These types of physical sources, such as light or magnetic fields, can penetrate the deep-sited targets and stimulate the liposomes, whereas minimally involving the tissues passed through by them (Weissleder, 2001). The liposomes sensitive to these types of physical sources undergo either immediate structural changes or indirect membrane disruption due to the thermal reaction to the source. Generally, liposomes react to electromagnetic waves *via* two mechanisms of photophysical and photochemical activations (Leung and Romanowski, 2012).

During the photo-physical mechanism, the liposomes lose their membrane integrity due to photo-thermal conversion without any chemical changes. Indeed, the energy of photons emitted by the light source is absorbed and converted to thermal/mechanical energies, which lead to the disordering of the liposome shell and the release of content (Chen et al., 2020). The photo-absorbers mediating the photo-thermal process include the molecular absorbers and plasmon resonant nanoparticles.

The lipophilic molecular absorbers could be integrated into the liposomes’ lipid bilayers while the hydrophilic absorbers are encapsulated into the liposomes. Once the light absorbers are irradiated by the source, they will act as a photo-thermal transducer and convert the light-to-heat effectively followed by releasing the content into the extra-liposomal environment (Ng et al., 2016). An early study showed that the calcein-loaded liposomes tended to release the content significantly under exposure to 488 and 577 nm laser lights. The photo-thermal release of the drug in the buffer was attributed to the photo-absorption of calcein at the 488 nm wavelength, while the photo-absorption of hemoglobin at 577 nm caused the drug release in blood samples (Khoobehi et al., 1990). An *in vivo* study combined an argon laser and adenosine diphosphate (ADP)-loaded liposomes showed the most efficient therapeutic gain of photocoagulation (Khoobehi et al., 1992). To visualize the release pattern of a thermo-sensitive liposome containing luciferin, transgenic reporter mice were administrated by the nano-carriers and then irradiated by a 527 nm laser. The *in vivo* bioluminescence imaging demonstrated that the liposomes immediately could release the molecular dye upon irradiation by the laser (Mackanos et al., 2009). On the other hand, some lipophilic dyes integrated into the liposome membrane showed a photolysis behavior after exposure to visible electromagnetic waves (Gregersen et al., 2010).

As another type of photo-thermal moieties, plasmonic nanoparticles can generate heat *via* harmonic oscillation of their free electrons triggered by the electric field of the electromagnetic waves. In addition to the great potential in enhancing CT image contrast (Cole et al., 2015) and radiotherapy gain (Zabihzadeh et al., 2018), the gold nanoparticles exhibit a significant plasmonic property in a wide range of wavelengths. Similar to the molecular absorbers, the gold nanostructures could be incorporated into the aqueous portion or on the surface of the liposomes. For instance, the gold-coated liposomes containing DOX showed a controllable manner in DOX release upon exposure to near infrared laser and caused to increase in cancer cell fatality (Wu et al., 2011). As for gold nanoparticles anchored on the liposome surface, the nano-hybrid carrier released about 70% of the encapsulated content while exposed to a 532 nm laser beam. The viability assay of breast cancer cells showed that DOX-loaded nanocarrier under laser beam irradiation significantly reduced cell viability (Qin et al., 2011). Although non-plasmonic gold nanoparticles were also proposed as photo-thermal agents to be integrated into the liposome structures, they mainly need a photon source with a shorter wavelength like UV and a long irradiation interval (Paasonen et al., 2007; An et al., 2010).

Also, liposomes release the encapsulated content *via* photochemical pathways using photo-sensitive lipid molecules embedded in the bilayer envelope. Photo-isomerization is one of the mechanisms in which light-sensitive moiety like azobenzene gives rise to conformational changes upon irradiation by a photon source (Chen et al., 2020). The azobenzene-based liposomes mainly activate at UV range source, limiting their clinical application due to the short penetration of UV electromagnetic waves into the body (Kano et al., 1981). More recently, a unique strategy was used to release the DOX under exposure to NIR-irradiation. They incorporated up-converting nanoparticles into DOX-loaded liposomes to convert the NIR beam to UV/blue light *in situ,* followed by photo-isomerization of azobenzene, destabilizing the membrane and releasing the drug (Yao et al., 2016).

On the other hand, the photo-cleavage mechanism triggers drug release from liposomes after exposure of photo-cleavable structures like plasmalogens to light sources. For example, a nano-system proved a photo-cleavage property during irradiation at 365 nm, followed by the release of penicillin (Goto et al., 2019). Moreover, photo-polymerization—the crosslinking of lipids under the incidence of photons—elevated the fluorescent agent release more than 100 times using a UV source (Bondurant and O’Brien, 1998). In a more recent study, liposomes equipped with a photo-sensitizer (verteporfin) experienced an adequate release of PAC1R antisense oligonucleotides under UV exposure due to the generation of ROS by verteporfin. The ROS act as a destabilizer of liposomes and endolysosomal membranes, where the release of the gene-silencing content subsequently decreases the PAC1R fluorescence intensity by 74% (Chen et al., 2017b).

Taking advantage of ultrasound waves, many studies have investigated the impact of mechanical waves on stimulating liposomes containing imaging or therapeutic agents. Thanks to the penetration of the ultrasound wave deeply and locally into the body while inducting low side effects, which extensively benefit patients in diagnostic and therapeutic procedures. Interestingly, the MRI-HIFU modality generates heating in the exposed site where thermo-sensitive nano-carriers like liposomes release their content (Zhou, 2011). Moreover, the system is capable to trace the drug biodistribution and pharmacokinetic pattern using MRI contrast agents embedded in the liposomes. For instance, a thermo-sensitive ultra-magnetic liposome was developed to carry an anti-vascularization agent (Combretastatin A4 phosphate). They showed that the thermo-responsive nano-carriers release the encapsulated content significantly upon exposure to HIFU and they significantly prohibited the tumor growth in CT26 tumor-bearing mice (Thébault et al., 2020). More recently, sono-sensitive liposomes-containing DOX were conjugated to monoclonal antibody Trastuzumab caused higher uptake in HER2+ breast cancer cells. The combination of the liposomes with low-frequency ultrasound showed a synergistic effect on cancer cell mortality (Elamir et al., 2021). In another study, gadolinium- and DOX-loaded liposomes were activated upon HIFU exposure, in which they release about whole encapsulated MRI contrast and anticancer contents. The MDA-MB-231 tumor-bearing mice treated with the liposomes and HIFU experienced a significant inhibition in tumor growth (Amrahli et al., 2021).

## Mesoporous silica nanoparticles

Among all different types of inorganic or hybrid organic/inorganic-based materials, silica is under the spotlight of research in nanomedicine. Silica is considered as a safe material for administration in food and cosmetics by US FDA (BernArdos and KouřimsKá, 2013). Silica NPs with several distinguishing features like easy functionalization during synthesis or postmodification and biocompatibility could be considered as potent nanocarrier for therapeutic purposes. Porous types of silica NPs have a higher surface area that makes them as an ideal adsorbent of molecules, ions, and therapeutic agents (Farjadian et al., 2015; Farjadian et al., 2017a; Farjadian et al., 2019c; Taqanaki et al., 2019; Mohammadi H. et al., 2020). Mesoporous silica nanoparticles (MSNs) with pore sizes between 2 and 50 nm have a tailorable structure with a high surface area and pore volumes and decoration possibility with various functional groups and have found distinguished applications as host of therapeutic agents (Farjadian et al., 2019c).

MSNs need a uniform particle size and pore volume large enough to enhance loading capacity and, if required, surface engineering properties on the external and internal surfaces need to be an ideal carrier for a drug. These parameters can be controlled during the synthesis by varying the reaction mixture’s pH, temperature, surfactant concentration, and silica source. The synthesis of MSNs occurs by the liquid crystal template mechanism wherein hydrolysis and condensation of silica could occur at the surface of surfactant micelles, and silica precursor (tetraethyl orthosilicate) transforms into solid silica (Narayan et al., 2018).

This section reports some examples of MSNs responsive to exogenous physical triggers and their application in the biomedical fields.

### Mesoporous silica nanoparticles in therapy and diagnosis

Within the unique properties of MSNs’ pores to be capped by gatekeepers, the possibility of designing smart engineered delivery agents becomes possible. MSNs have found diverse applications in medicine, catalysts, adsorbents, and sensors (Kankala et al., 2020).

MSNs utilization for cargo loading like small molecules (i.e., ions and drugs) and macromolecules such as proteins and genetic materials (i.e., DNA, RNA, siRNA, *etc.*), have made them a good platform for DDSs. Today, researchers are exploring oral and injection formulas of MSNs for therapeutic purposes. Farjadian et al. (2015) have introduced a novel approach to applying MSNs as an antidote agent for several toxicities.

Otherwise, by integrating metallic cores inside MSNs’ shells (metal@MSN), the MSNs with devoted properties of metal cores have been prepared (Zhang et al., 2021). Such novel innovations in preparing MSNs’ nanocomposites have allowed to utilization MSNs as a carrier of therapeutic agents with simultaneous imaging modalities in MRI and PET (Farjadian et al., 2019c). In this section, several types of MSNs used for therapeutic and imaging purposes regarding their response to physical stimulus are categorized and discussed.

### Temperature-responsive mesoporous silica nanoparticles

Among physically triggered therapeutic systems, the thermo-responsive strategy has been widely applied, especially in cancer therapy. MSNs could be engineered to deliver cargo in response to temperature changes, while modified with thermo-responsive polymers such as PNIPAAm. DDSs of this type are able to release drugs on the variation of temperature around their adjusted LCST. This system could be applied for efficient delivery through encountering temperature increase of the tumor site or for hyperthermia. As earliest model, Ibu delivery was investigated by mesostructured cellular foam modified by PNIPAAm inside nano-valves using the ATRP method (Wang T. et al., 2016). In an eco-friendly and straightforward route, PEO-*b*-PNIPAAm was utilized both as structure-directing and thermo-responsive agents while loaded with Ibu, and the system proved to have a thermosensitive release profile (Bathfield et al., 2016).

A multifunctional platform of MSN coated with thermos/pH-responsive polymers (pNIPAAm-co-pMA) was developed for co-delivery of paclitaxel, 5-Fu, cis-platin, and siRNA (targeting ABCG2) while also targeted with FA and the system was abbreviated as MSN@pNIPAAm-pMA/FA. The results demonstrated that both anticancer drug and siRNA were successfully delivered to CD133+ cancer cells by designed MSNs. *In vivo* studies showed that downregulation of ABCG2 parallel with enhanced efficiency of chemotherapeutic drugs and induced apoptosis in carcinoma cells of laryngeal (Qi et al., 2016). Another interesting example was reported for co-delivery of evodiamine and berberine (hydrophobic drugs) as an herbal Chinese medicine with synergistic antitumor activity. Lipid-coated MSN with thermo/pH polymeric coating (lipid-MSN@PNIPAAm-co-PMA) has proven to be an excellent smart carrier for the delivery of these two drugs *in vitro* and *in vivo* analyses.

A remotely targeted thermo/pH on-demand delivery and diagnosis system based on gold and magnetic NPs embedded silica nano-shuttles (MGNSs) coated with pNIPAM-co-pMA (MGNS@ PNIPAM-co-PAA) loaded with DOX was created, and the assembled structure was named as nano golf balls. Distinguished applications of the aforementioned structure were investigated through magnetic-field transport in; 1) glass capillary tubes as simulator of delivery through blood vessels, 2) porous hydrogels as simulator of human tissue (e.g., BBB, muscles, tendon, cartilage, and bone). The *in vitro* DOX release pattern was assessed in differentiated human neurons and epithelial HeLa cells (Wang D. et al., 2017).

The presence of diagnosable materials in the core of thermo-responsive MSNs has resulted in the formation of theranostic systems. MNP core-modified thermo-responsive agent coated with MSN (MNP@PNIPAAm-co-DMAEMA@MSN) was successfully created for MTX delivery and MR imaging.

MSN capped with paraffin showed to have a thermo-sensitive cargo release profile since paraffin could be melted at a certain temperature (Aznar et al., 2011). By this means AuNSts-coated MSNs were capped by paraffin for efficient DOX delivery through PTT (Hernández Montoto et al., 2018).

### Photo-triggered mesoporous silica nanoparticles for therapeutic and imaging

Light is one of the most powerful sources of exogenous stimuli and could be considered a safe treatment protocol (Li Y. et al., 2021). On the other hand, photo-responsive agents could be diagnosed through well-known imaging techniques such as photoacoustic, photothermal, and NIR (Shin et al., 2021). The intensity of photon sources could be easily tuned to visible, UV, and NIR by the sensitivity of therapeutic agents. Designing theranostic MSNs-based materials could be a step forward in facilitating the clinical trial requirements of these materials. NIR photon has been recognized as less risky with more efficacies in cells regarding UV and visible emissions (Chen et al., 2021). Several combinatorial techniques would provide MSNs to be photoresponsive. Several examples of such materials have been prepared through intra and/or extra pore modifications or gate capping with photo-responsive agents (Moodley and Singh, 2021). First reports in preparation of photochromism in a mesostructured silica appeared in early 2000. In these reports, several photochromic dyes like spiropyrane (Wirnsberger et al., 2000; Schomburg et al., 2001), azobenzene (Liu et al., 2003), and consequent photo controlled release was well performed by coumarin-modified MS material (Mal et al., 2003) were implemented.

Cyclodextrins (CD) with a self-assembled structure capable of host and guest interplay could potentially be applied as a gatekeeper of MSNs’ pores (Yi et al., 2018). Azo-benzene and related derivatives are recognized as photoresponsive agents, which possess their unique capability by cis and trans isomerization upon visible light and irradiation with UV light. Several combinations of CD with trans azo-benzene have been reported as photoresponsive gatekeepers of MSNs in drug delivery with the capability of gate opening while encountering UV irradiation through the formation of cis-isomer of azo-benzene with less CD interaction (Wang and Wu, 2016b; Zhao et al., 2017).

In recent years, some inorganic-based nanomaterials with emitting properties, including gold NPs, QDs (i.e., CdS, CuS,W_2_S, and Mo_2_S), and also organic-based fluorophores NPs like graphene QDs and carbon-dots with photo and thermal sensitivity have been applied as MSNs gatekeepers in designing DDSs (Table 3) (Wen et al., 2017; Yan et al., 2020). This part discusses some prominent types of such photoresponsive gatekeepers in combination with DDSs of MSNs. Theranostic MSNs with gold NPs and carbon QDs were applied for DOX delivery and showed highly trackable in cancer cell lines by fluorescence imaging (Akbarian et al., 2022). Gold-capped nanovalves of MSNs could efficiently act as photoresponsive switches for drug delivery. AuNPS has been demonstrated to be an efficient photoresponsive capping agent of MSN for the paclitaxel delivery system, which had revealed a “zero premature release” pattern before irradiation with a light source (Vivero-Escoto et al., 2009). Rod-shaped MSN with high cargo loading capacity and preferable cell internalization has been capped with gold nanorods (GNRs) for NIR photothermal therapy. Loading nano-pores with photosensitizer having an anticancer activity (Ce6) and irradiation source were performed by photodynamic therapy (PDT). Furthermore, the therapy was combined with dual-mode imaging by NIR fluorescence (NIRF) and photoacoustic (PA) (Sun et al., 2017).

**TABLE 3:** Physically triggered MSN concerning the type of stimulus and cargo for *in vitro* and *in vivo* investigation, therapy, and diagnosis.

MS-type	Cargo	Stimulus/therapy	Cellular assay-set	*In vivo* assay test	Imaging modality	Reference
IO@ST MSN	DOX	Light/PTT	**—**	**—**	**—**	Adam et al. (2022)
MSN-Azo-*β*-CD	Hexaconazole	Light (UV)	CCC-ESF-1	**—**	**—**	Upadhyay et al. (2021)
MSN-GQDs	RhB	Light/PTT	HeLa	**—**	**—**	Gao et al. (2019)
MoS2@MSN	Fluorogen PhENH2	Light/PTT	MDA-MB-231, HepG2		FI	Wang et al. (2019a)
PMO-CuS	DOX	Light/PTT	MDA-MB-231	S180 tumor	**—**	Cheng et al. (2018)
HMON-Mo-PMO	Mn_2_(CO)_10_	Light/PTT	U87MG	U87 MG tumor	PAI	Tang et al. (2018)
UCNPs@MS-POM-PEG	DOX	Light/PTT	Hela	Tumor xenograft	MRI, CT, and UCL	Xu et al. (2018a)
UCNPS@MSN	Merocyanine 540, OVA, TF	Light/PDT	CT26	CT26-tumor	**—**	Ding et al. (2018)
UCNPS@MSN-DNQ@*β*-CD	DOX	Light/PTT	HeLa	HeLa	**—**	Han et al. (2018)
				TBM		
UCNPS@MSN/MnO_2_	Ce6	Light/PDT	4T1	4T1 tumor	**—**	Gu et al. (2018)
MSN@PDA-AuNps	DOX	Light/PTT	**—**	**—**	**—**	Rahoui et al. (2018)
GNRs/PPy@MSN	DOX	Light/PTT	CCK8, CT26	CT26 TBM	**—**	Wang et al. (2018a)
GNRs@MSN-PUA	DOX	Light/PTT	Hela	**—**	**—**	
GNRs@MSN-*β*-CD-RLA	ICG	Light/PDT, PTT	MCF-7	MCF-7 TBM	IR-TI	Liu et al. (2018a)
AuNsts@MSN-parrafin	DOX	Light/PTT	HeLa	**—**	**—**	Hernández Montoto et al. (2018)
GNRS@MSN-	PFP	Light/PTT	A375	A375 TBM	US and PAI	Zhang et al. (2018a)
Au@MSN/HAP	DOX	Light/PTT	MCF-7	**—**	**—**	Song et al. (2018b)
Se@Au@MSN	DOX	Light/PTT	MCF-7-MDA-MB-231	MDA-MB-231 TBM	PTI	Zhao et al. (2018)
GNRs@MSN-Se-Se-FA	DOX and ICG	Light/PDT, PTT	HepG2	**—**	**—**	Ghamkhari et al. (2018)
ICG@HMSNs	DOX and DNA	Light/PTT	HeLa	HeLa cell tumor xenograft	**—**	Hai et al. (2018)
MSN@Bi_2_S_3_-RGD	DOX	Light/PTT	UMR-106	OS UMR-106 TBM	CTI	Lu et al. (2018)
CuS@MSN	DOX	Light/PTT	MDA-MB-231	HepG2 TBM	Photoacoustic and PET imaging	Wei et al. (2018a)
MSN-CuS/BSA	Ir-2 (Ir(III) complex)	Light/PDT, PTT	HeLa	Flank TBM	NIR FI, TI	Liu et al. (2018b)
QD@MSN-GO/FA	DOX	Light/PTT	HeLa	HeLa TBM	PAI	Song et al. (2018a)
MSN@PDA-GO	Cis-platin	Light/PTT	SH-SY5Y	**—**	**—**	Tran et al. (2018)
MSN-PEG	Pt(pyr), curcumin	Light	Skvo-3	**—**	**—**	Wu et al. (2018c)
MSN-*β*-CD-FA	Paclitaxel	Ultrasound	4T1	4T1 TBM	**—**	Wang et al. (2018b)
PMO	PB and Ce6	Light/PDT	HUVEC	U87MG	MRI and PA tomography	Yang et al. (2018c)
PMO	Porphyrin and siRNA	Light/PDT	MDA-MB-231	Zebrafish embryo	**—**	Mauriello Jimenez et al. (2018)
MSN@PDMAEMA	DOX, shRNA, and P-gp	Light	pG2/ADR	MDR solid tumor	**—**	Wu et al. (2018b)
MSN@PNIPAM-co-PAA-ICG	DOX	Light/PDT and PTT	HeLa	**—**	**—**	Falireas and Vamvakaki, (2018)
ROSP@MSN	DOX	Temperature	HeLa	**—**	**—**	Yu et al. (2018)
Gd MSN-ICG-Lip	DOX	Light/PDT, PTT	4T1	4T1 tumor	MRI, PAI, and NIR FI	Sun et al. (2018a)
UCNPS@MSN-Azo	DOX and Rose Bengal	Light/PDT	HeLa	**—**	**—**	Hou et al. (2017a)
UCNPS@MSN	DPA	Light/PTT	HeLa	U14 tumor	MRI and CTI	Lv et al. (2017)
MMSN@lipid-PEG	MTX and Zink phthalocyanine	Magnetic field and light/PDT	Hela and A549	HeLa tumor	FI and MRI	Liu et al. (2017a)
MSN@PPy-GQDS	MTX	Light/PDT	**—**	**—**	**—**	Liu et al. (2017b)
MMSN@GQDS	DOX	Light, magnetic field/PTT, and hyperthermia	4T1	**—**	**—**	Yao et al. (2017)
MSN-GO@DPA-HA	DOX	Light/PTT	HeLa	HeLa	FI	Shao et al. (2017)
MSN-cyanine	DOX	Light/PTT	4T1	4T1 tumor	NIR FI	Deng et al. (2017)
GNRs@MSN-HA-RGD	DOX	Light/PDT	Ovarian epithelial cell, and SKOV-3	**—**	**—**	Zhou et al. (2017)
MMSN@Au-PEG	-	Light/PDT	HePG2	**—**	MRI and CT	Hou et al. (2017b)
GRNs@MSN-CD-PGAE	DOX and pDNA	Light/PTT	Hek293 and C6	Glioma tumor	CT, PAI, and FI	Duan et al. (2017)
MSN-GNRs	Ce6	Light/PDT and PTT	4T1	4T1 tumor	PA and NIRF	Sun et al. (2017)
MSN- tLyP-1-WS2	DOX	Light/PTT	4T1	4T1 tumor	PTI	Lei et al. (2017)
rGO-PDA@MSN-HA	Ce6	Light/PDT	HCT-116, HT29, and NIH 3T3	**—**	**—**	Jiang et al. (2017)
Mn-UCNPs@MSN-PEG	DOX	Light	HeLa and L929	Axilla tumor xenograft	MRI, CT, and UCL	Xu et al. (2017)
GO@MS@mPEG-DSPE/FA	DOX	Light/PTT	MCF-7		**—**	Qin et al. (2016)
C-BON	DOX	Light/PTT	HeLa, MC3T3-E1 cells, and rMSC	Healthy female nude mice	NIRI	Singh et al. (2016b)
UCNP@MSN@*β*-CD	RhB	Light/PDT	A549 cells	**—**	**—**	Wang et al. (2016c)
MSN-Au nanosphere	DOX	Light	A2058 cells	Lung TBM	NIR and PET imaging and MRI	Cheng et al. (2016)
C-dots-Gd-MSN@pNIPAM-co-pMA	DOX and Ce6	Light/PTT and PDT	HeLa and L929 cells	U14 TBM	MRI and CT	Yang et al. (2016a)
MSNs-AuNBs-HA-azo-*β*-CD	DOX	Light/PTT	SCC cell spheroids	**—**	**—**	Chen et al. (2016)
MSN-BATA-BSA-PEG	DOX or G3-Pt and Ce6	Light	4T1, HeLa, and 293T	4T1 TBM	FI	Yang et al. (2016b)
PB@MSN-PEG	DOX	Light/PTT	MCF-7	MCF-7 tumor bearing mice	MRI, PAI, and IRTI	Su et al. (2016)
Ag@MSN/Au NFs	DOX	Light/PTT	HeLa	**—**	**—**	Tang et al. (2016)
UCNP@MSN@PEG/FA	Caged nucleic acid, ZnPc, and MC540	Light/PDT	B16-F0	B16-F0 and C57BL/6 MBT subcutaneous melanoma	**—**	Gnanasammandhan et al. (2016)
MSN-β-CD-Azo	DOX	Red light	**—**	**—**	**—**	Wang and Wu, (2016a)
PMO-Cy5.5	DOX	Light/PTT	MDA-MB-435 and MCF-7	Healthy ICR mice	NIR FI	Lu et al. (2016)
EuGdOx@MSF	DOX	Light/PTT and PDT	HeLa cells	B16F0 MBT tumors	MRI, PTI, and FI	Kalluru et al. (2016)
MSN@ PDMAEMA-perylene	DOX	Light	MCF-7	**—**	**—**	Wang et al. (2016a)
MMSN@lip	DOX	Magnetic field	MCF7 and U87	**—**	**—**	Sharifabad et al. (2016)
MCM48-Gd@AuNC, MCM41-Gd@AuNC	DOX	Light	SKOV3	**—**	**—**	Niu et al. (2014a)
MMSN-FA	CMP	Magnetic field	HeLa	**—**	**—**	Sahu et al. (2014)
MSN-DNA-CuS	DOX	Light/PTT	HeLa	**—**	**—**	Zhang et al. (2015)
UCNPs@MS@α-CD	DOX	Light	HeLa	**—**	**—**	Cui et al. (2015)
MMSN-CdS	CMP	Light	CHO	**—**	**—**	Knežević and Lin, (2013)
AuMS-dsDNA	DOX and siRNA	Light	HeLa	**—**	**—**	Chang et al. (2012)
Au-PMO	DOX	Light	MCF-7	**—**	**—**	Croissant et al. (2014)
MSN- galactose	CMP and porphyrin derivatives	Light/PDT	Capan-1, HCT-116, and MDA-MB-231			Gary-Bobo et al. (2012)
MSN-[Ru]	Amsacrine	Light	HeLa	**—**	**—**	Knežević, (2013)
MMSN-FA	CMP	Magnetic field	PANC-1 and BxPC3	**—**	**—**	Liong et al. (2008)
UCNPs@MSN-Azo	DOX	Light/PTT	HeLa	**—**	**—**	Liu et al. (2013b)
Lipid-MSN@PNIPAAm-co-pMA	Evodiamine and berberine	Temperature	HeLa, HepG2,HCT-29	Tumor embeded by EMT-6 cells	**—**	Feng et al. (2019)
MMSNs@PNIPAAm-co-PNHMA	DOX	Temperature and magnetic field		EL4 murine lymphoma	**—**	Guisasola et al. (2018)
MNP@PNIPAAm-co-DMAEMA@MSN	MTX	Temperature	A549	**—**	MRI	Farshbaf et al. (2018)
MSN@PNIPAAm-co-PAA	Ibu	Temperature	**—**	**—**	**—**	Jin et al. (2018)
MGNS- PNIPAAm-co-PAA	DOX	Temperature and light/PDT	HeLa and NPCs	**—**	**—**	Wang et al. (2017a)
MSN-PEG-PCL	DOX	Temperature	A549	**—**	**—**	Cho et al. (2017)
UCNPs@Ce6@MSN		Temperature and light/PDT	MDA-MB-435	MDA-MB-435 tumor-bearing nude mice	MRI	Zeng et al. (2016)
GNSC	DTX	Temperature and light/PTT	B16-F10	B16 tumor bearing cells	**—**	Wang et al. (2016b)
MMSN@CPS and MMSN@HP	Fluorecein sodium	Temperature				Guisasola et al. (2016)
MSN@PNIPAAm	Ibu	Temperature				Brunella et al. (2016)
MSN@pNIPAAm-co-BVIm	Cytochrom C	Temperature	MCF-7	**—**	**—**	Eltohamy et al. (2016)
MMNP	**—**	Magnetic field and temperature	LNCaP	LNCaP TBM and HDFn human skin fibroblast	SWIFT MRI	Hurley et al. (2016)
MMNP	DOX	Magnetic field and temperature	HeLa	**—**	**—**	Yuan et al. (2016)
MSN@PNIPAAm-co-pMA/FA	Cis-Pt, PTX, 5FU, and SiRNA	Temperature	HepG2	HepG2 cells TBM	**—**	Qi et al. (2016)
MSN@PEO-b-PNIPAAm	Ibu	Temperature	**—**	**—**	**—**	Bathfield et al. (2016)
Au NRs@MS@p(NIPAAm-co-BVIM)	DOX	Temperature and NIR/PTT	HeLa cells	**—**	CT	Baek et al. (2016)

HMSN, hollow mesoporous silica; IO@ST MSN, iron oxide@stellate MSN; Ag@HMSN@ PNIPAAm-co-AA, silver NPs as core with MSN layer and p (NIPAAm-*co*-acrylic acid); MSN-GQDs, MSN with graphene quantum dot; MoS2, molybdenum disulfide embedded MSN; lipid-MSN@pNIPAM-co-pMA, lipid-coated MSN modified with pNIPAM-co-polymethacrylic acid; PMO-CuS, copper sulfide-capped mesoporous periodic organosilica; HMON-Mo-PMO, hallow mesoporous organosilica nanopartivle (HMON) modified with Mo(VI)-polyoxometalate (POM); DNQ, 2-diazo-1,2-naphthoquinones; MMSNs, magnetic MSNs; PNHMA, poly N-hydroxymethylacrylamide; MSN@PDA-AuNps, gold-modified polydopamine coated MSNs; MNP@PNIPAM-co-PDMAEMA@MSN, magnetic nanoparticles (MNP) coated with PNIPAM-co-poly (N,N′-dimethylaminoethylmathacrylamide (DMAEMA) conjugated to MSN; GNRs/PPy@MSN, gold nanorods (GNRS)/polypyrrole (PPy) coated MSN; PUA, poly(urethane-amine); RLA, peptide RLA ([RLARLAR]2); AuNsts@MSN-parrafine, gold nanostars coated with MSNs capped with paraffin; GNRS@MSN, gold nanorattles-coated MSN; PFP, perflouropentene; Au@MSN/HAP, gold nanoparticle-coated MSN hybrid hydroxyapatite (HAP); Se, selenium; Se–Se, diselenide derivatives; ICG@HMSNs, indocyanine green-loaded hallow MSN (HMSN); CT, computed tomography; MSN@Bi2S3-RGD, MSN coated with bismuth sulfide conjugated to arginine-glycine-aspartic acid (RGD) peptide; BSA, bovine serum albumin; OVA, ovalbumin; TF, tumor cell fragment; QD@MSN-GO/FA, quantum dot-modified MSN coated graphene oxide (GO) conjugated folic acid (FA); PEG, polyethyleneglycol; MMSN-CdS; MMSN gated cadmium sulfide (CdS); MSN-*β*-CD-FA, MSN gated with cyclodextrineconjugated FA; PB, Prussian blue; Ce6, chlorin e6; PAA, polyacrylic acid; ROSP, ROS-modified polymer; GdMSNs-ICG-Lip, Gd-dopped MSN conjugated with ICG loaded with liposomes; Azo, azobenzene; HA, hyaluronic acid; MGNS, magnetic and gold embedded silica nanoshuttles; PCL, poly(*β*-caprolactone); CD-PGEA, *β*-CD-modified poly (glycidyl methacrylate); MSN- tLyP-1-WS2, tumor homing/penetrating peptide-modified tungsten disulfide; GNSC, gold nanoshell capsule; C-BON, carbon dot-generated bioactive organosilica nanospheres; rMSC, rat mesenchymal stem cell; MMSN@CPS, MMSN with crosslinked polymer shell; MMSN@HP, MMSN with hairy polymer; TCPP, tumor-targeting cellular membrane penetrating peptide; TPP, tumor-targeting therapeutic peptide; AuNBs, gold nano-bipyramids; BVIM, 3-vinyl imidazolium bromide; MSN-BATA-BSA-PEG, MSNs-coated BSA *via* bis-(alkylthio)alkene (BATA) linker modified with PEG; Ag@MSN/Au NFs, silver nanoparticles coated with MS with gold nanoframes (NFs); PMO-Cy5.5, cyanine 5.5 conjugated PMO; and EuGdOx@MSF, lanthanide-doped MS frameworks.

One of the well-known types of QDs NPs is CuS, with low-cost availability and low toxicity, which has found applications in PTT due to intrinsic capability in NIR adsorption. An exciting paradigm of CuS-capped MSNs was reported as a controllable DOX delivery in PTT. The CuS NPs were conjugated to nanopores through double-stranded oligonucleotide sequences (MSN-DNA-CuS) that could be de-hybridized in encountering localized heat (Zhang et al., 2015). Another example is the CuS capped to yolk/shell structure of periodic mesoporous organosilica (PMO-CuS) NPs that provided triple-responsive nanoplatforms with efficient performance in the DOX delivery system *via* PTT (Cheng et al., 2018).

CdS also applied as a gatekeeper in designing a photoresponsive camptothecine delivery system of MNP-coated MSN (*via* carbamate photoresponsive linkage) (Knežević and Lin, 2013). Carbon dot (C-dot)-capped MSN was a powerful fluorescent agent in designing DDS with bioimaging capability (Jiao et al., 2016).

Mo_2_S-embedded MSN has exhibited to be an excellent PT agent and while fabricated with fluorogen PhENH_2_ and targeted with folic acid (FA) showed to be a potent agent in targeted PTT and fluorescence imaging (Wang J. et al., 2019). A novel PT-inducing developed capping agent of MSN is graphene quantum dots (GQDs) that successfully practiced in the rhodamine B delivery system (Gao et al., 2019). Other examples include GQDs-capped MMSN for PTT and magnetic hyperthermia in the DOX delivery system (Yao et al., 2017). Bismuth sulfide (Bi_2_S_3_) NPs have also been recognized as a NIR light absorbing and contrast agent of CT imaging. Recently, two novel types of theranostic light-responsive MSNs systems with Bi_2_S_3_ have been reported (Li L. et al., 2018; Lu et al., 2018). MSN-coated Bi_2_S_3_ targeted with trastuzumab (Bi_2_S_3_-MSN-trastuzumab) loaded with DOX was created for simultaneous PTT and CT imaging (Li L. et al., 2018). Another system was explicitly designed for osteosarcoma by conjugating RGD (Bi_2_S_3_-MSN-RGD) to the system for DOX delivery and malignant tumor PTT (Lu et al., 2018).

Other developments in the establishment of light-responsive nano-carriers lay in the formation of core/shell structure of common responsive elements even with MSN as a core (MSN@Shell) or in the opposite, MSN as a shell (core@MSN). Metallic-based NPs with emitting properties have played a distinct role in designing theranostic agents. In this regard, gold-coated MMSNs (MNP-coated MSN) were designed for PTT and dual MR and CT imaging (Hou X. et al., 2017). Polydopamine-gold nanoparticle was applied as a shell for MSN (MSN@PDA-Au), proved to be an effective DOX delivery system triggered by NIR, and showed to be potent in PTT (Rahoui et al., 2018). On the other hand, gold NPs (AuNPs) and gold nanorods (GNRs) have been efficiently applied as the core of MSNs.

**FIGURE 6:**
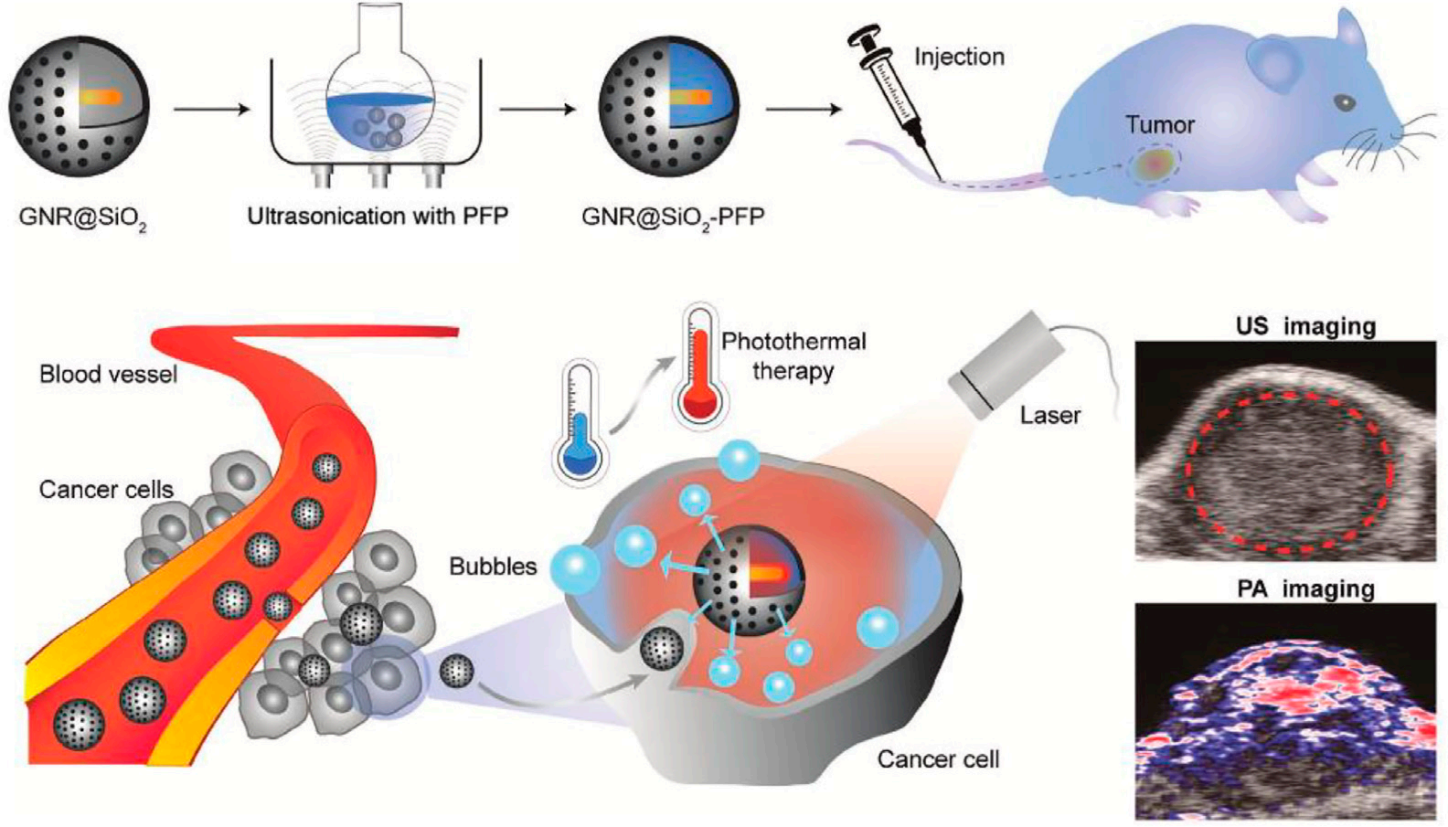
Schematic illustration of GNRS@MSN filled with PFP from synthesis, *in vivo* injection, cell entrance, PTT, and dual-mode imaging (US and PA). Reprinted with permission from Wiley (Li C. et al., 2018).

A dual imaging nanotheranostic system based on biodegradable gold nanorattle (GNR)-coated MSN filled (GNRs@MSN) with perfluoropentane (PFP) was developed for melanoma PTT and ultrasound (US) and photoacoustic (PA) imaging. A schematic illustrating synthesis, *in vivo* injection, and PTT and dual US/PA imaging are presented in Figure 6. Upon NIR irradiation, the GNRs generated heat that induces PFP to have a liquid/gas phase transition, which resulted in nano-bubbles formation. Nano-bubbles convert to microbubbles that can improve EPR and enhance signals of US (Li C. et al., 2018). Several other examples of photo-responsive AuNPs-based MSN systems are presented in Table 3.

Newly established up-converted NPs (UCNPs), known as an unusual combination of lanthanide ions, showed to be potent photo-responsive agents due to their capacity in adsorbing NIR and changing to UV or visible emission (Escudero et al., 2016). UCNPs could be employed as the core of MSN as an NIR-responsive system and detected through fluorescence (Wang et al., 2011). Several examples of such systems are presented in Table 3.

Multifunctional luminescent-based UCNPs of NaYF_4_:Yb^3+^/Er^3+^ with MSN coating and thermoresponsive capped were developed for DOX delivery (Zhang et al., 2013). Multi-color emitted MSN designed UCNPs@MSN-Azo-coated UCNPs ((NaYF_4_: Yb, Tm) @ 0.6(NaYF_4_: Yb, Er) capped with azobenzene and loaded with RB and DOX was developed for DDs *via* PDT. The UCNPs capable of emitting UV: blue and green: red multi-band light was acted as a stimulus of light-responsive Azo and photo-sensitizer of RB. The mechanism of action of this multifunctional nano platform in blood vessels is depicted in Figure 6. After the accumulation of NPs in the tumor site due to the EPR effect, the NIR light source was excited with the potential of deep penetrating that could activate UCNPs. The green emission could be adsorbed by RB and produce ROS for PDT. At the same time, UV/blue emission would activate Azo to open the gates for DOX release (Hou B. et al., 2017).

### Magnetic field responsive mesoporous silica nanoparticles

Magnetic responsive MSN-based materials, with MNP in their composition, are recognized as powerful carriers for efficient delivery systems and utilization in MR imaging. Several theranostic systems have been developed based on the MNP core and MSN shell (MMSN). Some examples of these potent systems are discussed here. Multifunctional superparamagnetic iron oxide-based MSN with dibenzo-crown ethers (MMSN-crown ethers) periphery with trimodal responsiveness to pH/ultrasound and magnetic field was developed for DOX delivery. Ultrasonic waves could stimulate sodium or cesium ions binding with crown ethers with pH-responsive binding that act as gatekeepers of the system. *In vitro* MRI analysis showed the high relaxivity of the NPs in the magnetic field, potent theranostic agents (Lee et al., 2013).

A nanoassembly of MMSN coated with chitosan (charge converted polymer-g-FA and citraconic anhydride) targeted by TAT peptide (MMSN@CS/TAT) and loaded by CMP as DNA-toxin antitumor drug was established. MRI observed a high accumulation of NPs in the tumor site. Upon this phenomenon, the FA acts as a target for receptor-mediated endocytosis in the cancer cells. Through endocytosis into the lysosomes, the polymeric layer charges’ reversed from negative to positive and separated from MMSN, and TAT peptide assisted the carrier entrance to nucleus, where CMP could induce apoptosis (Li et al., 2014). A novel microwave-triggered system for etoposide targeting *via* external magnetic field was created based on magnetic/Fe_3_O_4_ core and zinc oxide/ZnO interlayer coated by mesoporous silica (MNP@ZnO_2_@MS). The ZnO interlayer could act as an absorber of microwave irradiation with an excellent thermal response. These magneto-responsive particles showed sustained control release upon microwave irradiation (Qiu et al., 2014). A maghemite core MSN capped with 1-tetradecanol (TD) molecule (MMSN-TD) as a heat-triggered agent was developed for delivery of DOX. This system showed to be diagnosable *via* MRI, and a plausible mode of release was established through TD gate-keeping at 40°C (Cho et al., 2017). A multifunctional magneto-responsive MSN was developed for multimodal imaging (PAI, MRI, and FI) and chemo and photodynamic therapy. By this mean, Fe_3_O_4_ as core with MSN shell and protective layer of lipid-PEG and lipid-PEG-MTX was created (MMSN-lipid-PEG-MTX) while loaded with DOX and zinc phthalocyanine (ZnPc) (MMSN-lipid-PEG-MTX/DOX&ZnPc) as a photosensitizer. The presence of lipid-PEG-MTX in periphery of MMSN was effective in increasing dispersion stability and preventing drug leakage while decreasing hemolytic activity. The magnetic core provided magneto targeting to the tumor site, and the MTX conjugate would selectively kill cancer cells by overexpressing folate receptors. ZnPc was loaded in the lipid layer and served as a fluorescence tracking agent in FI and a source of PDT ROS.

## Magnetic nanoparticles in therapy and diagnosis

The original idea of using magnetic particles to deliver medicinal agents to a specific body area was formed in the late 1970s. This method injects the whole drug/magnetic carrier into the body through venous or arterial vessels (Kodama and materials, 1999). Then, by applying an external magnetic field and creating a magnetic gradient in a specific body area, a set of drugs/magnetic carriers are transported to that location (motor site) by the circulation and accumulation (Meyer et al., 2001). When the accumulation of particles in the motor site is well performed, the therapeutic agents (drugs) are separated from their magnetic carrier and released at the tumor site (Shubayev et al., 2009). Drug-releasing mediators can include enzymatic activation, changes in physiological conditions such as changes in ambient pH, changes in osmolality (the degree of solubility of a solution per unit of solvent), or changes in ambient temperature (Allen and Cullis, 2004). Physical triggers in releasing medical agents from MNPs are mostly temperature and magnetic fields due to MNPs hyperthermia and magnetic properties (Kauscher et al., 2019). Other stimuli are less important for these particles. This means, in this part of the review we discuss hyperthermia and magnetic therapy. Furthermore, MRI is the most important imaging modality for these particles (Farjadian et al., 2017b).

Targeted drug delivery is more relevant to cancer treatment because the main challenge in cancer treatment is to target and kill cancer cells so that they have as little effect on healthy cells as possible (Bae and Park, 2011). One of the aims of nanotechnology is to put drugs on carriers (NPs), then send, and release them into specific cells, which is called targeted drug delivery. By using magnetic nanoparticles and generating a magnetic field, the drug can be intelligently delivered to the desired tissue without damaging other tissues (Shen et al., 2013).

Diagnosis of the disease in the early stages is essential for improvement and treatment methods. Iron oxide nanoparticles are currently the only magnetic nanomaterials used in clinical medicine as a contrast agent in MRI and as a carrier in drug delivery (Dadfar et al., 2019). These particles have also found applications in separating cancer cells through functionalization with antibodies (Haghighi et al., 2019; Haghighi et al., 2020). Experiments performed on iron oxide nanoparticles over the years show that these particles have no immediate or long-term toxic effects *in vivo*, and the presence of some nanoparticles with nanocarriers enhances their effect on cancer cells (Liu G. et al., 2013).

### Temperature-triggered magnetic nanoparticles

Cancer treatment is one of the most critical challenges, facing medical knowledge and drug delivery. Because conventional cancer treatments such as chemotherapy, surgery, and radiation are not very effective in treating some cancers such as glioblastoma, hyperthermia can be considered a safe approach to the definitive treatment of such cancers (Chang et al., 2018). Treatment of cancer by induced excitation of biocompatible superparamagnetic nanoparticles by intermittent magnetic field heating of specific organs or tissues to temperatures around 41–47°C to treat cancer is called hyperthermia (Chang et al., 2021). The hyperthermia process with magnetic ferrofluid suggests the possibility of specific heat localization; cancer cells are more sensitive to temperature than normal cells (Das et al., 2019). Superparamagnetic nanoparticles can cause excess heat to be transferred to the target area through oscillations of magnetic moments within the nanoparticles (Xiao and Du, 2020). Thus, solid cancer cells will be killed while normal tissue cells will remain at a temperature below 41–47°C. The heating potential depends to a large extent on the size and shape of the particles. Therefore, using single-order magnetic particles in nanosized is preferred over multi-order particles in micro size (Ha et al., 2018). Because nanoparticles can withstand alternating magnetic fields, the amount of heat generated varies depending on the frequency, the intensity of the magnetic field, and the time it takes to be in the field. In this case, after the particles are placed inside the cells, and then the body is placed in a magnetic field, these particles produce localized heat in the cancerous mass and accelerate the destruction of the tumor (Behrouzkia et al., 2016). Therefore, hyperthermia will effectively increase the treatment of solid cancer (Cole et al., 2011). To wrap up all data on magnetic approaches in the clinical sector, here are some recently published articles (Table 4). For most of them, doxorubicin has been considered as cargo; however, some of them did not include any extra medicinal cargo, just by magnetic-mediated hyperthermia trying to fight the target, cancer cells. To better explain the recent contents, mentioning some references in the table would be helpful. In the study by Huang et al. (2020), gelatin was used as a matrix to encapsulate doxorubicin. Then the complex was covered by the outer layer of alginate and magnetic iron oxide particles (core-shell nano-system).

**TABLE 4:** Physically triggered MNP concerning the type of stimulus and cargo for *in vitro* and *in vivo* investigation, therapy, and diagnosis.

Magnetic type	Cargo	Stimulus/therapy	Cellular assay set	*In vivo* test	Imaging modality	Reference
Gelatin/Fe_3_O_4_–alginate	DOX	Magnetic field	MCF-7	—	FI	Huang et al. (2020)
IO	DOX	Magnetic field	HT29	—	FI	Augustin et al. (2016)
IO NPs/loaded starch-octanoic micelles	DOX	Magnetic field	BEL-7402	Hepatic carcinoma - mice	Real-time FI	Jie et al. (2019)
IO-carboxymethylcellulose	DOX	Magnetic field	U87	—	Confocal laser scanning microscopy	Carvalho et al. (2019)
GO/IO/curcumin–HSA	DOX	Magnetic field	SH-SY5Y	—	—	Lerra et al. (2018)
IO-PEG- ICG	DOX	Light/PTT	CCK8 and CT26	C6 glioma-bearing rats	MRI	Shen et al. (2019)
FA@MSN@Fe_3_O_4_	Erlotinib	—	HeLa	—	—	Avedian et al. (2018)
MTX-CSC@MNPs	Erlotinib	—	OVCAR-3	—	—	Fathi et al. (2020)
GEM-MNP-pHLIP	GEM	Magnetic field	PANC-1	—	MRI	Han et al. (2020)
PEG-CS-IONPs-Cy5.5	MTX	Magnetic field	Hela	BALB/C nude mice and adult Sprague–Dawley rats	MRI and FI	Lin et al. (2015)
PEG-CS-IONPs	MTX	Magnetic field	MCF-7	—	—	Karimi et al. (2017)
Fe_3_O_4_//chitosan	Telmisartan	Magnetic field	PC-3	—	—	Dhavale et al. (2021)
NiFe_2_O_4_/PEG/lipid–polymer	Zidovudine	Magnetic field	SK-BR-3	—	FI	Joshy et al. (2020)
Porous carbon-coated MNPHA	DOX	Light/PTT	HeLa and HUVECs	HeLa cell tumor-bearing mice	MRI	Wu et al. (2019)
IO Nanocubes	DOX	Temperature	A431	Athymic immunodeficient xenograft mouse	IR	Mai et al. (2019)
Dextran@ MNP@ PVC-Co-PVI	5-Fu	Temperature and pH	MCF-7	—	—	Anirudhan et al. (2020)
MNP@HAP	—	Temperature	MG-63 osteosarcoma	—	—	Mondal et al. (2017)
MNP+MgO	—	Magnetic fluid hyperthermia	U87MG	Xenografted rat model	FI	Jung-tak et al. (2018)
MNP@PMMA	—	Magnetic hyperthermia	MB-231	MB-231 human breast cancer xenograft in nude mice		Ling et al. (2017)
MNP@tetraganeth gum@PAA	DOX	Magnetic field	HeLa	—	—	Sayadnia et al. (2021)
IO@PEI	RNA		U-118MG	—	MRI	Grabowska et al. (2021)

MCF-7, Michigan Cancer Foundation-7; U87MG, Uppsala 87 malignant glioma; FI, fluorescence microscopy imaging; MG-63, osteoblast-like human osteosarcoma cell line; 5-FU, fluorouracil; IR, infrared; HUVECs, human umbilical vein endothelial cells; MNP, methylmethacrylate; IO, iron oxide; PMMA, polymethylmethacrylate; HAP, hydroxyapatite; PEI, polyethyleneimine; HAS, human serum albumin; and PANC-1, pancreatic adenocarcinoma cell line.

However, the size was relatively large (401 nm), after 6 hours of the synthesized particles reaching the nucleus of MCF 7 cancer cells. Then, after 12 h, it was observed that the cell death rate was reduced by 50%. In another study by Baek et al. (2016), a three-component system was used. In the system’s center, gold nanoparticles were included around the nucleus by DOX-containing MSN designed as drug reservoirs. Then a temperature-sensitive gate (poly(N-isopropylacrylamide)-based N-butyl imidazolium copolymer) was used to finalize the invention. In addition to labeling, gold particles in this design produce heat under NIR, which eventually leads to the opening of the gates and release of the drug. Another study was about the synthesis pH-responsive system (Carvalho et al., 2019).

Other studies have focused on other forms of matter. In the study of Ling et al., polymethylmethacrylate (PMMA)-Fe_3_O_4_ in the form of liquid-to-solid transition state was used. This system will be able to suppress the MB-231 breast cancer xenograft model in mice from the injection route in the liquid state. It was seen that after injecting this system, the cancer site was considered with high accuracy, without showing any leakage in other locations (Ling et al., 2017). Other cases of research have followed a more or less similar pattern. In Anirudhan and Christa’s study, glycidyl methacrylate grafted dextran was used to coat iron oxide nanoparticles and N-vinylcaprolactam and N-vinylimidazole monomers were used to create temperature and pH sensitivities. After studying the drug release at different acid strengths, it was found that the designed system was able to follow a release pattern similar to the cancerous environment (Anirudhan et al., 2020).

## Nanopharmaceuticals on the market

In the end, it is necessary to mention some of the nano-systems that have been able to enter the field of treatment. Among the types of nanoparticles mentioned so far, nanocrystals, liposome and lipid-based nano-systems, polymer-based nanoparticles, protein-based nano-systems, and metal-based nanopharmaceuticals have been successful in the field of therapy (Farjadian et al., 2019a). The following Table 5 introduces this category and mentions some examples.

**TABLE 5:** List of approved nano-systems in market and therapeutic industries.

Nano-system	Example	General comment	Reference
Metal based	Feraheme^®^	This medicine is prescribed for patients who experience iron accumulation. Its approval dates back to 2009 by the FDA. Although it has been seen that the amount of 510 mg of it has been completely tolerated for adults, but a series of negative side effects have been observed, including hypotension, diarrhea, dizziness, and constipation	McCormack, (2012)
Protein based	Abraxane^®^	Abraxane is NP consisting of albumin protein conjugated with PTX. The size of these particles usually reaches 130 nm and is very important in controlling and managing breast cancer	Gradishar et al. (2005)
Polymer based	Cimzia^®^	This nanoparticle consists of a Fab fragment attached to a PEG. The FDA approved it in 2008, and it is used in treating many patients such as ankylosing spondylitis, Crohn’s disease, and psoriatic arthritis. This nano-system explicitly attacks the TNF-α through its protein part and leads to the inhibition	Nesbitt et al. (2007)
	Adagen^®^	Like Cimzia, this nano-system also consists of a protein part (adenosine deaminase) and a PEG fragment. This medicine is used when the patient’s body suffers from a lack of adenosine deaminase production	Murguia-Favela et al. (2020)
	Neulasta^®^	With the PEGylation of filgrastim protein, this nano-system entered the therapeutic field (in 2002). Neulasta is a stimulator of leukocyte proliferation in diseases such as consequent infections arising from a lack of neutrophils and febrile neutropenia. It has been seen that the blood circulation time of filgrastim alone is between 3.5 and 3.8 h, while it increases to 42 h with the form conjugated with PEG.	Alconcel et al. (2011)
Liposome and lipid-based	Doxil^®^	This exciting drug is PEGylated liposomes that have trapped the effective drug DOX inside. Due to the extensive use of DOX drug in a wide range of cancers, Doxil is also used in different types of cancers such as metastatic ovarian cancer and AIDS-related Kaposi’s sarcoma (KS). The year of its approval by the FDA dates back to 1995. This drug is available in different sizes, between 80 and 90 nm. Like many liposome drugs, this engineering aims to increase blood circulation time in the body, which results in the use of smaller amounts and doses in the body. With this strategy, the possible effect of side effects is also minimized	Farjadian et al. (2019a)
	Onivyde^®^	Like the previous case, this drug is the liposomal form of the effective irinotecan drug, which has been used in pancreatic cancer. It has been seen that the use of this drug with other anticancer agents increases its effect (synergistic effect)	Zhang, (2016)
	Ostim^®^	This crystalline nano-system with a diameter of 20 nm is composed of calcium hydroxyapatite [Ca_10_(PO_4_)_6_(OH)_2_]. As a scaffold for bone growth in uses such as dentistry and bone repair, it has entered the field of treatment since 2004	Brandt et al. (2010)
Nanocrystals	Rapamune^®^	This drug is used more in cases with a history of kidney transplant rejection. The active part of this nano-system, which is a type of immune system inhibitor, is a macrocyclic triene, an antibiotic extracted from *Streptomyces hygroscopicus* bacteria. After years of use to prevent kidney transplant rejection (since 2010), in 2015 with FDA approval, this drug was also used in the lymphangioleiomyomatosis disease	Kesisoglou et al. (2007), Bobo et al. (2016)

## Conclusion and future outlook

The key properties of physical stimuli-responsive therapeutic/diagnostic systems compared to the direct delivery of therapeutic agents are their ability to release the drug in a stimuli-responsive manner, high drug loading capability, lower toxicity, synergistic therapeutic efficacy, and biocompatibility. Table 6 compares the types of physical triggers and the advantages and disadvantages of such strategies. The proper nanocarrier design and structural optimization are crucial challenges to physical stimuli-responsive nano drug delivery systems. Multiple lines of evidence indicate that it mainly relies on empirical analysis, such as the type of disease, the characteristics of the drugs, and the nanocarrier physicochemical properties. These areas required further investigation and evaluation. As for future perspectives, the data that would be achieved from these extensive and comprehensive studies lead to the development of more well-designed physical stimuli-responsive nanoparticles capable of reaching the market, providing a novel generation of nanocarriers more suitable for clinical application with minimum adverse side effects and maximum efficiency.

**TABLE 6:** Advantages and limitations of physically triggered systems for therapy and diagnosis.

Physical trigger	Advantage	Limitation
Temperature	• Easy availability	• Possibility of scorching
	• Low cost	• No control over termination
	• Applicable in personalized medicine	
Light	• High spatiotemporal targeting	• Superficial penetration
	• It could be used as both therapeutic and diagnostic	• Skin damage by the use of UV and other short wavelengths
	• It has opened its way to trade better than other strategies	
	• They can be considered for photodynamic and photothermal therapies	
	• The existence of many light-sensitive materials	
Magnetic fields	• Deep penetration into tissues	• Safety of this method is still controversial
	• High spatiotemporal targeting	• Lack of transdermal applications
	• Controlling the cell/tissue mechanobiology	
	• Enriched magnetic hyperthermia	
Ultrasound	• High spatiotemporal targeting	• Harmful at high ultrasound power
	• Appreciable tissue penetration	• Not suitable for lung therapy
	• Useful in drug delivery to the brain	

Another promising approach is to focus on nanoparticles with the potential to respond to multiple triggers. For instance, the light source has a high point focusing accuracy but is very poor at penetrating tissue. On the other hand, ultrasound waves have the best permeability to the tissue, but they are weak to have the accuracy of targeting like light. It is possible to generate a system with the positive points of both origin systems and synergistically plays significant strengthening effects in the treatment process by combining these two physical systems. Although physical stimuli-responsive nanoparticles are primarily evaluated for preclinical applications, they will shortly be an essential part of the clinician’s armory in addressing drug delivery and diagnosis.

## Glossary

5-FU: 5-fluorouracil
ADR: adriamycin
AgNPs: silver nanoparticles
Alg: alginate
ATRP: atom transfer radical polymerization
AuNPs: gold nanoparticles
Azo: azobenzene
BDPI: pyrrole-substituted iodinated BODIPY
BSA: bovine serum albumin
C 102: Coumarin 102
CD: cyclodextrin,
CdS: cadmium sulfide
Ce6: chlorin e6
Chol: cholesterol
cis-Pt: cis-platin
ClAlPc: chloroaluminum phthalocyanine
CMC: critical micelle concentration
CMP: camptothecin
CNTs: carbon nanotubes
CPT: camptothecin
CS: chitosan
CT: computed tomography
DDSs: drug delivery systems
DFM: dark-field microscopy
DOX: doxorubicin
DTX: docetaxel
EAPs: electric active polymers
EPR: enhanced permeation and retention
FA: folic acid
FDA: Food and Drug Administration
FI: fluorescence imaging
GEM: gemcitabine
GNP: gold nanoparticle
GO: graphene oxide
GQDs: graphene QDs
HA: hyaluronic acid
HMSN: hallow MSN
Ibu: ibuprofen
ICG: indocyanine green
IONP: iron oxide nanoparticles
IR780: photoresponsive NIR dye
LCST: lower critical solution temperature
MMSN: MNP core with MS shell
MNP: magnetic nanoparticles
MRI: magnetic resonance imaging
MS: mesoporous silica
MSN: mesoporous silica nanoparticles
MTX: methotrexate
NGs: nanogels
NIR: near-infrared
NIRI: near-infrared imaging
NPs: nanoparticles
NR: Nile Red
NRs: nano-rattles
NRS: nano-rods
Nsts: nano-stars
OI: optical imaging
OVA: ovalbumin
Ox: oxide
OXA: oxaliplatin
PAA: polyacrylic acid
PAAm: poly acrylamide
PAI: photoacoustic imaging
PAS: poly aspartic acid
PB: prussian blue
PCL: poly(caprolactone)
PDA: polydopamine
PDMAEMA: poly(2-dimethylaminoethyl methacrylate)
PDT: photodynamic therapy
PEG: polyethyleneglycol
PEI: polyethyleneimine
PEO: polyethylene oxide
PHEMA: poly(2-hydroxyethylmethacrylate
PHPMA: poly(N-hydroxypropyl)methacrylamide
PLA: poly(lactic acid)
PLGA: polyglycolic acid
Plys: poly(l-lysine)
PMAA: poly(methacrylic acid)
PMs: polymeric micelles
PNIPAAm: poly(N-isopropylacrylamide)
PNVCL: poly(N-vinylcaprolactam)
PpIX: protoporphyrin IX
PPO: polypropylene oxide
PPy: polypyrrole
PS: polystyrene
PTNS: physically triggered nano-systems
PTT: photothermal therapy
PTX: paclitaxel
Py: pyridine
PyF: pyropheophorbide-a
QDs: quantum dots
RAFT: reversible addition-fragmentation chain transfer polymerization
RB: Rose Bengal
rGO: reduced graphene oxide
RhB: rhodamine B
ROP: ring-opening polymerization
ROS: reactive oxygen species
SHG: second harmonic generation
SN38: camptothecin analog
SNA: spherical nucleic acid
SPECT: single-photon emission CT
TBM: tumor-bearing mice
TI: thermal imaging
TNF: tumor necrosis factor
UCST: upper critical solution temperature
UPCNS: up-conversion NPs
USI: ultrasound imaging
UV: ultra violet
Vis: visible
ZnPc: zinc phthalocyanine
